# An Integrative Multi-scale Analysis of the Dynamic DNA Methylation Landscape in Aging

**DOI:** 10.1371/journal.pgen.1004996

**Published:** 2015-02-18

**Authors:** Tian Yuan, Yinming Jiao, Simone de Jong, Roel A. Ophoff, Stephan Beck, Andrew E. Teschendorff

**Affiliations:** 1 Key Laboratory of Computational Biology, CAS-MPG Partner Institute for Computational Biology, Chinese Academy of Sciences, Shanghai Institute for Biological Sciences, Shanghai, China; 2 Center for Neurobehavioral Genetics, Los Angeles, California, USA; 3 Medical Genomics Group, UCL Cancer Institute, University College London, London, United Kingdom; 4 Statistical Genomics Group, UCL Cancer Institute, University College London, London, United Kingdom; Albert Einstein College of Medicine, UNITED STATES

## Abstract

Recent studies have demonstrated that the DNA methylome changes with age. This epigenetic drift may have deep implications for cellular differentiation and disease development. However, it remains unclear how much of this drift is functional or caused by underlying changes in cell subtype composition. Moreover, no study has yet comprehensively explored epigenetic drift at different genomic length scales and in relation to regulatory elements.

Here we conduct an in-depth analysis of epigenetic drift in blood tissue. We demonstrate that most of the age-associated drift is independent of the increase in the granulocyte to lymphocyte ratio that accompanies aging and that enrichment of age-hypermethylated CpG islands increases upon adjustment for cellular composition. We further find that drift has only a minimal impact on in-cis gene expression, acting primarily to stabilize pre-existing baseline expression levels. By studying epigenetic drift at different genomic length scales, we demonstrate the existence of mega-base scale age-associated hypomethylated blocks, covering approximately 14% of the human genome, and which exhibit preferential hypomethylation in age-matched cancer tissue. Importantly, we demonstrate the feasibility of integrating Illumina 450k DNA methylation with ENCODE data to identify transcription factors with key roles in cellular development and aging. Specifically, we identify REST and regulatory factors of the histone methyltransferase MLL complex, whose function may be disrupted in aging.

In summary, most of the epigenetic drift seen in blood is independent of changes in blood cell type composition, and exhibits patterns at different genomic length scales reminiscent of those seen in cancer. Integration of Illumina 450k with appropriate ENCODE data may represent a fruitful approach to identify transcription factors with key roles in aging and disease.

## Author Summary

Two well-known features of aging are the gradual decline of the body’s ability to regenerate tissues, as well as an increased incidence of diseases like cancer and Alzheimers. One of the most recent exciting findings which may underlie the aging process is a gradual modification of DNA, called epigenetic drift, which is effected by the covalent addition and removal of methyl groups, which in turn can deregulate the activity of nearby genes. However, this study presents the most convincing evidence to date that epigenetic drift acts to stabilize the activity levels of nearby genes. This study shows that instead, epigenetic drift may act primarly to disrupt DNA binding patterns of proteins which regulate the activity of many genes, and moreover identifies specific regulatory proteins with key roles in cancer and Alzheimers. The study also performs the most comprehensive analysis of epigenetic drift at different spatial scales, demonstrating that epigenetic drift on the largest length scales is highly reminiscent of those seen in cancer. In summary, this work substantially supports the view that epigenetic drift may contribute to the age-associated increased risk of diseases like cancer and Alzheimers, by disrupting master regulators of genomewide gene activity.

## Introduction

Recent studies, using Illumina Infinium beadarrays, have demonstrated that genome-wide DNA methylation patterns change with age [1–6]. Further studies have indicated that this age-associated epigenetic drift may have deep implications for stem-cell biology [7], disease development [8] and possibly also human evolution [9, 10]. Thus, it has become of great interest and importance to study the detailed dynamics of the DNA methylation landscape in response to aging.

In this regard however there are many pressing unanswered questions. First is the issue of cellular heterogeneity. Although a number of studies have already indicated that a significant component of the epigenetic drift is tissue-independent, and therefore unlikely to be caused by underlying changes in cell subtype composition [2, 3, 6], this result remains unproven and contrasts with a number of Epigenome-Wide Association Studies (EWAS) for specific diseases, notably Rheumatoid Arthritis [11] and cancer [12, 13], which have shown that in the disease context, correction of intra-sample cellular heterogeneity can be critical [11, 14]. Indeed, a number of statistical methods have emerged allowing correction for cell subtype compositional changes [15, 16]. However, to date no study has applied these algorithms in the context of ageing to assess how much of the epigenetic drift is due to underlying changes in cell-type composition. This is particularly pertinent in the context of blood tissue, since in this tissue it is known that there is an age-associated increase in the granulocyte to lymphocyte ratio [12, 15].

Another outstanding issue concerns the characteristic genomic length-scales of epigenetic drift. Cancer epigenome studies have demonstrated that cancer-associated DNA methylation changes exhibit differential patterns at different length scales, with local hypermethylation at CpG islands (CGIs) often immersed within large-megabase scale blocks of widespread hypomethylation [17, 18]. However, so far no study has comprehensively explored whether hypomethylated blocks emerge in normal tissue as a function of age, and whether these bear any resemblance to those seen in cancer or early neoplastic lesions. Given that age is a major risk factor for many diseases, specially cancer, the existence of such blocks could provide an important indicator of future disease development.

Finally, epigenetic drift is thought to be one mechanism underlying the decline of stem-cell function with age, thus compromising normal homeostasis [7, 9]. That this might be the case is further supported by a recent study of dynamic DNA methylation changes during cellular development [19], which showed that differentially methylated regions (DMRs) in development are strongly enriched for regulatory elements. Indeed, differential binding of transcription factors as a result of differential DNA methylation at transcription factor binding sites (TFBSs) may be an important mechanism of cellular development and lineage specification. It follows that age-associated epigenetic drift may compromise binding of key lineage-specifying transcription factors. Although there is already considerable evidence that developmental transcription factors represent targets of epigenetic drift [2, 3], no study has yet explored in detail which transcription factor binding profiles may be disrupted in aging as a result of drift.

More generally, the ability to detect putative differential binding of transcription factors by studying DNA methylation patterns around their TFBSs is specially important because, unlike ChIP-Seq, DNA methylation can be reliably measured genome-wide from limited amounts of DNA [20]. Thus, DNA methylation can be measured in large numbers of clinical or non-clinical specimens, allowing in principle transcription factors disrupted in disease and ageing to be identified [21]. Moreover, differential binding caused by differential DNA methylation may represent a more accurate way of assessing differential activity of transcription factors. For instance, assessing transcription factor activity from its mRNA or protein expression level is problematic due to postranslational modifications [22]. Thus, DNA methylation may provide improved or complementary information about transcription factor activity.

In this work we perform a comprehensive study of DNA methylation drift addressing the core issues mentioned above. We focus on blood because of its availability and the fact that the largest studies to date have been performed in this tissue [4, 5]. By using a powerful algorithm to correct for cellular heterogeneity [15], we demonstrate that most of the epigenetic drift, specially the age-hypermethylated component of it, is not caused by changes in blood cell subtype composition. We further demonstrate that age-associated DNA methylation patterns exhibit spatial patterns at different genomic length scales which are reminiscent of those seen in cancer. Moreover, by integrating large-scale 450k DNA methylation data with extensive TFBS information from the ENCODE project [23–25], we demonstrate the feasibility of the Illumina 450k technology to identify important lineage-specific transcription factors. Having demonstrated the feasibility of this approach in the context of cellular development, we next apply the same method to aging, identifying novel transcription factors which may be implicated in the aging process.

## Results

### Overall patterns of age-associated DNA methylation are independent of changes in blood cell subtype composition

Age-associated DNA methylation changes have so far only been studied comprehensively at the level of individual CpGs [1–6], and in whole blood tissue, a tissue for which the largest sample collections are available [4, 5]. However, the interpretation of age-associated changes in whole blood is problematic due to underlying age-associated changes in blood cell subtype proportions [12, 15]. To address these challenges, recent studies have developed methods that allow more robust inference of differential DNA methylation at the level of genomic regions [26, 27] and which is independent of underlying changes in cell subtype composition [15, 16].

**Fig 1:**
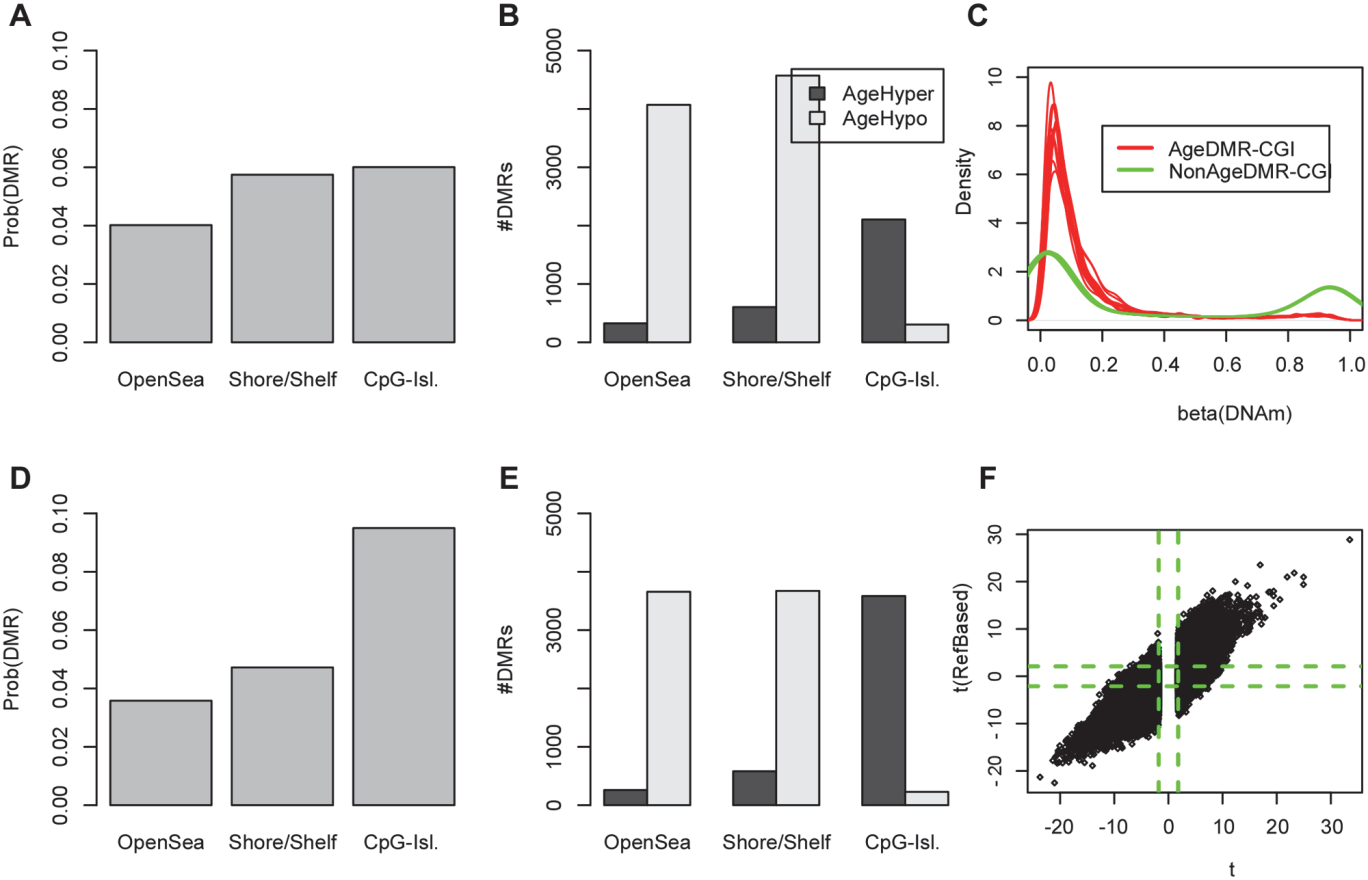
Age-DMRs are mostly enriched for CGIs and the enrichment of age-hypermethylated CGIs increases after adjustment for changes in blood cell subtype composition. **A)** Probabilities that a randomly picked open-sea, shore/shelf and CpG-island region is an age-DMR (defined as the top 5% of DMRs). **B)** Relative numbers of age-hypermethylated and age-hypomethylated DMRs within each regional class. **C)** Focusing on CpG-islands, density plots of beta DNAm values of 2500 age-DMRs (red) vs. 2500 non-age associated DMRs (green) (*P* > 0.8). **D)** As A) but now for age-DMRs derived using the reference-based method of Houseman et al, which adjusts for putative changes in blood cell subtype proportions. **E)** As B), but now for the adjusted analysis. **F)** Scatterplot of the t-statistics of age-DMRs (defined here as those with FDR < 0.05 in the unadjusted analysis) (x-axis) against their corresponding t-statistics from the reference-based adjusted analysis (t(RefBased), y-axis). Green dashed lines indicate the lines of significance at FDR < 0.05.

Here we decided to use one of these recent methods [15], to re-analyze one of the largest Illumina 450k DNA methylation data sets available, encompassing whole blood samples from 656 individuals spanning a wide age range (19 to 101 year olds) [5]. In order to call differential methylation more robustly and to avoid any statistical biases caused by neighboring probes on the beadarray, we decided to collapse neighboring probes in the genome into specific clusters [26], dividing all probes up into 3 different regional classes: CpG islands, shelves & shores and open sea. Specifically, probes within a class and which were spatially close were grouped into regions with an upper bound of 1.5Kb set on the size of these regions (Methods). This resulted in 239650 regional clusters, consisting of 109424 open sea, 90090 shelve/shore, and 40136 CpG island regions. Probe methylation values within regions were averaged. Supervised linear regression analysis was performed for each of the 239650 regions, adjusting for plate and gender (plate was fully correlated with ethnicity in such a way that adjusting for plate also adjusts for ethnicity (Methods)), which identified 124352 age-DMRs at an FDR < 0.05. Focusing on the top 5% age-DMRs (a total of 11982), the far majority (75%) were hypomethylated with age, consistent with previous observations [4, 5]. Although the majority of the top 5% DMRs fell into open sea and shore/shelf regions, CpG-islands were more strongly enriched given their overall lower numbers in the genome (Fig. 1A). We observed a marked difference between open sea or shore/shelf regions and CpG islands, with approximately 90% of open sea/shore/shelf age-DMRs exhibiting hypomethylation, in contrast to age-DMRs mapping to CpG islands, which were overwhelmingly hypermethylated (87%) with age (Fig. 1B). We verified that all these results remained unchanged had we used a less stringent Bonferroni correction threshold (26019 age-DMRs) (**S1_Fig in** S1 Text). Age-DMRs mapping to CpG islands predominantly exhibited low levels of DNA methylation in the youngest individuals of our cohort (age range 19–28), whereas CpG-islands whose DNAm did not change with age exhibited both low as well as high levels of DNAm (Fig. 1C).

In order to assess the impact of changes in blood cell subtype composition, we applied the reference-based Houseman algorithm [15] to estimate the relative proportions of 6 blood cell subtypes (CD4+ & CD8+ T-cells, NK-cells, B-cells, monocytes and granulocytes) in the 656 whole blood samples. The algorithm predicted an age-associated decline in the relative numbers of T and B-cell lymphocytes, whilst the proportion of granulocytes and monocytes increased (**S2_Fig in** S1 Text), in line with previous observations [7, 12]. By using these sample-specific cell proportion estimates as covariates in the linear regressions, we rederived an adjusted set of age-DMRs, which resulted in 85299 regions at FDR < 0.05, i.e 69% of the total number identified without adjustment. Focusing on the top 5% (11982 regions) age-DMRs from the adjusted analysis, we observed that the enrichment of CpG-islands among the adjusted age-DMRs was increased relative to the unadjusted analysis and relative to open-sea and shore/shelf regions (Fig. 1D–E). Among the top 5% age-DMRs from the adjusted analysis, 63% and 37% were hypo and hypermethylated, respectively, i.e. less hypomethylation was observed after adjustment for cell-type composition, consistent with previous data [7, 12]. Importantly, we observed a strong correlation between the unadjusted and adjusted analysis, with approximately 50% of the unadjusted age-DMRs (FDR < 0.05) retaining significance at the same FDR level in the adjusted analysis (Fig. 1F). Using a more stringent threshold, i.e declaring only the top 5% of regions as age-DMRs in the unadjusted analysis, resulted in 83% of these passing an FDR < 0.05 in the adjusted analysis. Thus, these results indicate that although less hypomethylation is observed after adjustment, that most of the changes, and in particular those involving hypermethylation, are independent of blood cell subtype, in agreement with previous observations made with the older Illumina 27k technology [2, 3, 28].

### Age-associated hypomethylated blocks exist, but only a subset are enriched for age-associated CGI hypermethylation

**Fig 2:**
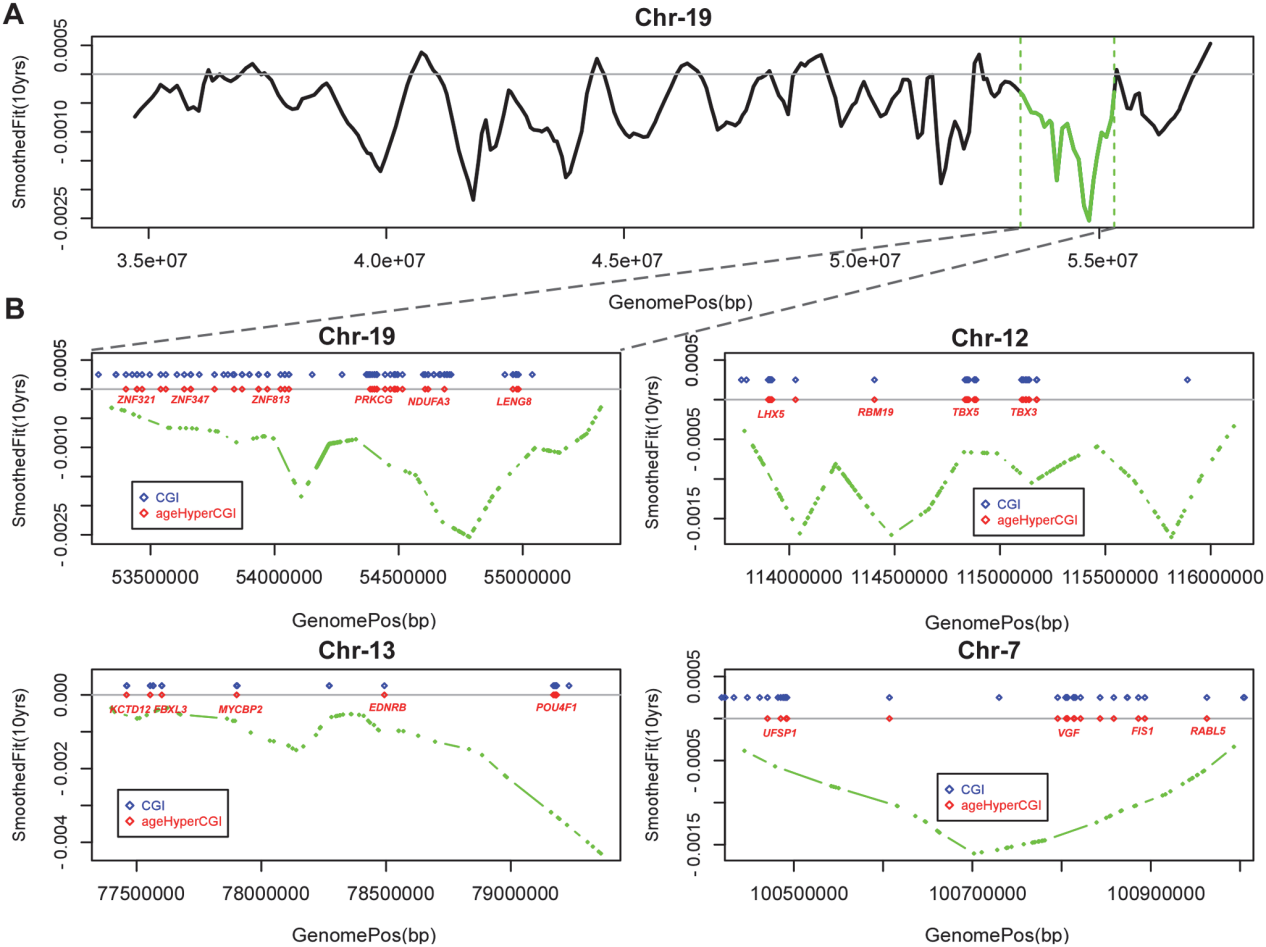
Age-associated hypomethylated blocks enriched for hypermethylated CGIs. **A)** Example of a large genomic region on chromosome-19 containing a significant age-associated hypomethylated block (indicated in green). y-axis gives the fit from the Bumphunter algorithm indicating the methylation change for an increase in 10 age-years, x-axis the genomic position. **B)** Selected age-hypomethylated blocks enriched for CGIs undergoing hypermethylation. Each plot shows the fit from the Bumphunter algorithm indicating the methylation change per 10 age-years (y-axis) as a function of opensea probe position (x-axis). In blue we indicate the positions of CGIs, and in red those that are significantly hypermethylated with age. Some of the genes associated with the age-hypermethylated CGIs are indicated in red.

It is of interest to study epigenetic drift on larger genomic length scales, since recent studies have demonstrated the existence of large mega-base scale blocks of hypomethylation in disease [17, 18, 26]. We sought to determine if such hypomethylated blocks are also seen in normal tissue as a function of age. To this end, we applied the same block-finding algorithm of Aryee et al [26]. We identified a total of 351 age-associated blocks (FWER < 0.05), with an overall genome coverage of 433Mb (i.e. 14% of the human genome) and with a median block size of 1.2Mb (**Table S1 in** S1 Text). The overwhelming majority (98%) of these blocks exhibited hypomethylation (**Table S1 in** S1 Text, Fig. 2A). Next, we asked if age-hyperM DMRs (FDR < 0.001) mapping to CGIs were enriched within these blocks. Most blocks (309/351, 88%) were either not enriched for age-hyperM CGI-DMRs or the numbers of age-hyperM CGIs were too small to reliable assess statistical significance (**Table S1 in** S1 Text). Of the 42 blocks which were enriched (Binomial *P* < 0.05), 18 showed strong enrichment encompassing at least 10 age-hyperM CGI-DMRs (Table 1). We note that because blocks are not directly comparable, adjustment for multiple testing in this context is prone to substantial error. However, using Benjamini-Hochberg, 21 of these 42 blocks remained significant at an FDR < 0.05, and all 42 were significant at a more relaxed adjusted threshold of FDR < 0.17. Importantly, the bi-modality of enrichment of age-associated hypomethylated blocks indicates that the mechanisms leading to DNA methylation deregulation may be distinct for different genomic blocks.

**Table 1:** 18 Age-associated hypomethylated open sea blocks enriched for CGIs undergoing age-associated hypermethylation, and containing at least 10 such CGIs.

Chr	Length(Mb)	Probes	FWER(hypoM)	Obs(AgeHyperCGI)	Exp(AgeHyperCGI)	P(Enr)
16	4.88	204	< 0.002	36	28.20	0.034
19	1.97	181	< 0.002	38	24.42	**6e-04**
13	4.23	133	< 0.002	10	4.65	**0.001**
5	2.02	112	< 0.002	13	7.56	**0.007**
2	1.24	79	< 0.002	16	9.01	**0.002**
2	2.27	88	< 0.002	12	6.69	**0.005**
12	2.32	135	0.002	38	14.83	**3e-12**
13	1.96	63	0.002	11	6.98	0.025
14	1.58	51	0.002	12	5.23	**2e-04**
4	2.08	27	0.002	11	6.40	0.011
4	0.70	15	0.002	10	5.52	0.008
6	3.18	188	0.004	21	12.79	**0.003**
2	2.28	94	0.004	20	13.66	0.017
20	1.97	77	0.004	32	22.97	0.011
5	2.58	113	0.006	10	4.07	**2e-04**
8	1.91	60	0.008	20	7.85	**2e-07**
7	1.22	50	0.028	25	18.32	0.026
7	0.55	51	0.034	17	9.01	**7e-04**

Columns label chromosome, length of block (Mb), number of contiguous open sea regions defining hypomethylated block, the family wise error rate (FWER) for the hypomethylated block, observed number of age-hyperM CGIs within block, expected number of age-hyperM CGIs within block and Binomial P-value of enrichment of age-hyperM CGIs within block. In bold-face we indicate those significant under adjustment for multiple testing (Benjamini-Hochberg FDR < 0.05).

Many of the hypomethylated blocks contained many well separated CGIs exhibiting hypermethylation and targeting multiple genes (Fig. 2B). Among these was a block on chromosome-19 containing multiple genes encoding for zinc-finger proteins, as well as a block on chromosome-13 containing *MYCBP2* and *POU4F1*. However, we also observed hypomethylated blocks where the enrichment was driven by hypermethylated CGIs which were all in close proximity to each other, for example this was the case for a block on chromosome-4 with all CGIs in the neighborhood of the *HAND2* gene (**S3_Fig in** S1 Text), a gene which has already been linked to aging [1] and which has also been causally implicated in endometrial carcinogenesis [29]. Thus, our analysis suggests that only a relatively small fraction of hypomethylated blocks are enriched for age-hypermethylated CGIs, with a few of these blocks representing hotspots of CGI hypermethylation.

### Age-associated hypomethylated blocks exhibit preferential hypomethylation in cancer, independently of age and tissue

**Fig 3:**
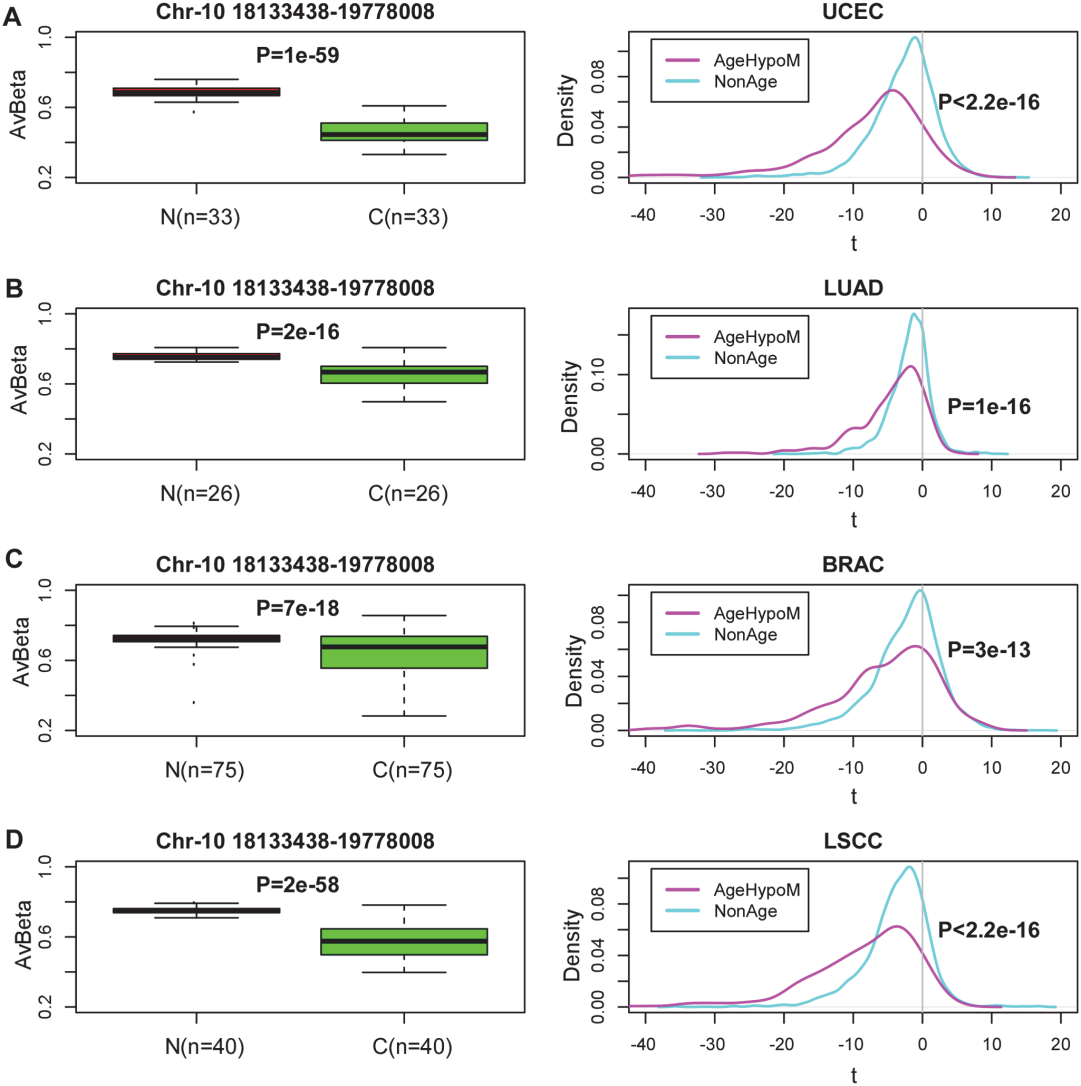
Age-associated hypomethylated blocks exhibit preferential hypomethylation in cancer. Left boxplots show the average beta methylation levels over open-sea regions within an age-associated hypomethylated block on Chr-10 in normal tissue and age-matched cancers from the TCGA. (**A)** Endometrial Cancer (UCEC), **B)** Lung Adenoma Carcinoma (LUAD), **C)** Breast Cancer, **D)** Lung Squamous Cell Carcinoma (LSCC). P-values in boxplots are from a Wilcoxon rank sum test. Number of samples in each group is indicated. Right panel depicts the density distribution of the t-statistics of all age-hypomethylated blocks (magenta), as assessed between normal and age-matched cancer tissue. The cyan curve represents the corresponding statistics for a random set of non-age associated blocks, matched for number and length distribution of the observed age-hypomethylated blocks. P-value is from a Kolmogorov-Smirnov test.

Next, we asked if age-associated hypomethylated blocks overlap significantly with those seen in cancer. To investigate this, we mapped the age-associated hypomethylated blocks onto cancer DNA methylation data from the TCGA [30], comparing average DNA methylation levels of open sea probes within blocks between normal and age-matched cancer tissue. Blocks showed significantly lower DNAm levels in cancer tissue compared to age-matched normal samples, independently of tissue type (Fig. 3). Next, we randomly picked open sea blocks which did not show significant age-associated hypomethylation in blood and recomputed statistics of differential methylation for these blocks. Comparing the distribution of the statistics of differential DNAm for the age-associated blocks to these randomly picked blocks, revealed significantly larger negative statistics (Kolmogorov-Smirnov *P* < 1*e* − 10) for the age-associated hypoM blocks (Fig. 3). Thus, this extends previous observations made at the level of CGIs to the mega-base scale block level, further supporting the view that much of the cancer-associated deregulation of DNA methylation may already be present in aged normal tissue [31].

### Most age-DMRs do not affect expression of target genes

Next, we decided to shed further light on the potential functional effect of epigenetic drift. Although a recent study, which performed matched Illumina 450k and gene expression profiling for the same blood samples, concluded that most drift does not cause gene expression changes, this study was significantly underpowered [32]. Thus, given that another unmatched study did report a weak association between age-DMRs and gene expression [5], the functional significance of epigenetic drift remains unclear. To address this question, we analysed one of the largest whole blood gene expression datasets available, encompassing over 200 samples [33] (Methods). We focused on DNAm levels in CpG island probe clusters with probes mapping to within 200bp of the transcription start site (TSS) or 1st exon, since it was shown previously that Illumina 450k probes mapping to these regions provide the best predictive power of a corresponding gene’s expression level [34]. However, we did not find any global statistical significance between the statistics of differential DNA methylation and those of differential expression (**S4_Fig in** S1 Text).

**Fig 4:**
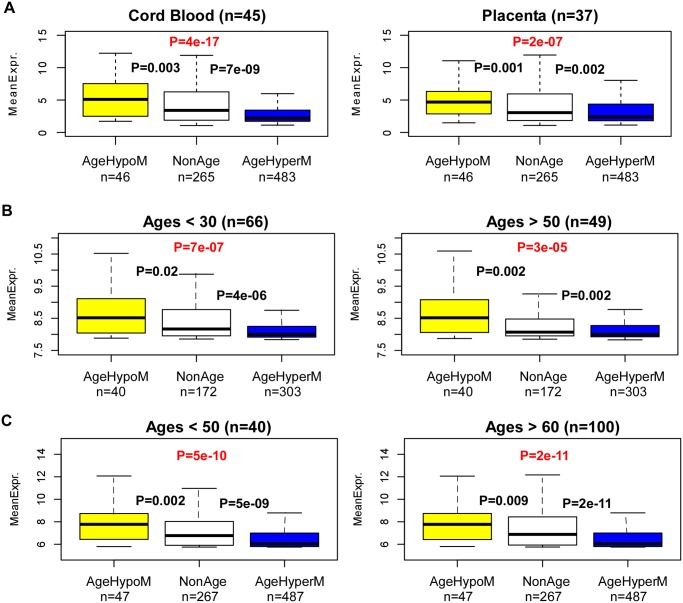
Age-associated hypermethylation (hypomethylation) targets lowly (highly) expressed genes. **A)** Boxplots of mRNA expression levels in cord blood and placenta samples, for three sets of genes: (i) genes undergoing age-associated hypomethylation in their CGI promoters (AgeHypoM), (ii) genes not undergoing any age-associated DNAm changes (NonAgeDMR), and (iii) genes undergoing age-associated hypermethylation in their CGI promoters (AgeHyperM). P-values shown between groupds are from a Wilcoxon-rank sum test comparing the respective neighboring gene classes. The number of genes in each class is indicated below plot. The P-value from a linear regression of expression against gene-class is also indicated in red. **B)** Left panel: As A) but for the whole blood samples of de Jong et al 2014, for individuals under the age of 30. Right panel: As left panel, but now for 49 whole blood samples from people over the age of 50. **C)** As B) but now for the expression data set of Beineke et al 2012.

To understand why, we posited that age hyperM CGI-DMRs may preferentially target genes which are normally not expressed in blood, whilst age-hypoM CGI-DMRs may correspondingly target more highly expressed genes. To test this hypothesis it is important to first obtain estimates of the baseline levels of expression in blood, ideally at birth. To this end, we obtained an expression data set of cord blood and placenta samples [35]. Confirming our hypothesis, we observed a highly significant trend with genes undergoing age-hypermethylation around their TSSs exhibiting significantly lower levels of expression than genes exhibiting age-associated hypomethylation (Fig. 4A). Thus, age-associated modulation of DNA methylation is unlikely to cause widespread *in-cis* expression alterations, because the direction of DNAm change may only act to stabilize pre-existing expression levels. To validate this result, we decided to repeat this analysis in the de Jong et al cohort [33], separately on young and old age groups. Remarkably, we observed a similar trend as in the cord blood and placenta data (Fig. 4B). Importantly, we can also observe no difference in the expression levels of age-hyperM or age-hypoM genes between the younger and older individuals (Fig. 4B).

In order to validate these results further, we collected another relatively large gene expression data set of 198 whole blood samples, albeit this cohort consisted of significantly older individuals [36]. Confirming our earlier result, we did not find that genes mapping to age-hyperM DMRs exhibited age-associated decreases in gene expression, and similarly that genes mapping to age-hypoM DMRs did not exhibit gene expression increases (**S4_Fig in** S1 Text). Importantly, in this cohort we also observed a significantly higher level of expression of genes undergoing age-associated hypoM in their promoters compared to those undergoing age-associated hyperM, a result which was also independent of age-group (Fig. 4C).

### Integration of Illumina 450k DNA methylation and ENCODE data to identify transcription factors associated with cellular phenotypes

A recent study used whole-genome bisulfite sequencing (WGBS) to demonstrate that differential DNA methylation can be a powerful means of identifying regulatory elements, including transcription factors, which play key roles in cellular development [19]. Similarly, we posited that differential DNA methylation changes associated with age, if enriched for transcription factor binding sites (TFBS), may allows us to identify transcription factors whose differential binding and activity patterns become disrupted with age.

**Fig 5:**
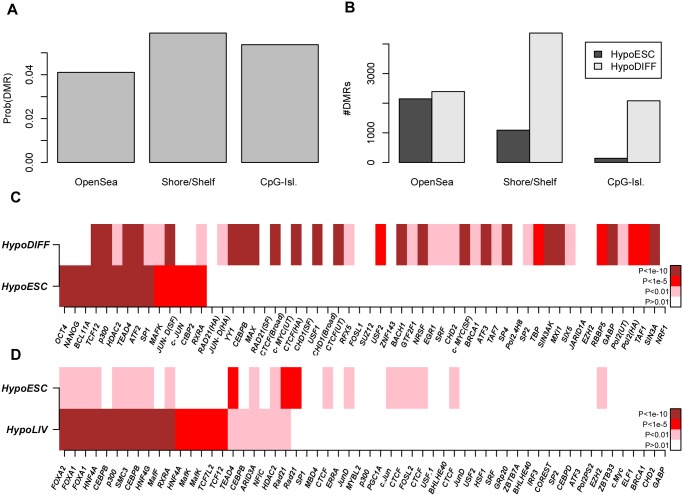
Identification of pluripotent and lineage-specific TFs by integration of Illumina 450k DNAm with ENCODE data. **A)** Probabilities that a randomly picked open-sea, shore/shelf and CpG-island region is a DMR between pluripotent and differentiated cells (defined as the top 5% of DMRs). **B)** Relative numbers of DMRs hypomethylated within hESCs and differentiated cell types and within each regional class. **C)** Enrichment heatmap of transcription factor binding sites (as assessed in H1-hESC) for 58 TFs, among the DMRs hypomethylated in hESCs (HypoESC) and DMRs hypomethylated in differentiated cells (HypoDIFF). **D)** Enrichment heatmap of transcription factor binding sites (HepG2 line) for the top ranked 58 TFs, among DMRs hypomethylated in hESCs (HypoESC) and DMRs hypomethylated in liver cells (HypoLIV). In C-D), TFs have been ranked according to the significance of enrichment among HypoESC DMRs. Color codes: white (*P* > 0.01), pink (*P* < 0.01), red (*P* < 1*e* − 5), brown (*P* < 1*e* − 10). ChIP-Seq binding profiles of the same TF but generated by different labs are distinguished by an abbreviation of the corresponding lab: SF (Stanford), HA (Hudson-Alpha).

In order to assess the potential of the Illumina 450k platform to identify relevant transcription factors through differential DNAm patterns, we first considered the case of cellular development and lineage specification, where key transcription factors are already known. Specifically, we collected an Illumina 450k DNA methylation data set of 153 samples, encompassing human embryonic stem cell (hESCs) and induced pluripotent stem cell (iPSCs) lines, as well as somatic (differentiated) tissue specimens (Methods) [37]. We derived DMRs between the hESCs and the differentiated cell types from a total of 244347 regional clusters (Fig. 5A). Focusing on the top 5% of DMRs (all passed FDR < 0.001), we observed that the majority were hypomethylated in differentiated cells, with open sea regions generally exhibiting hypomethylation, in contrast to shore/shelf and CGIs which were mostly hypermethylated (Fig. 5B). To assess enrichment of TFBS among the DMRs, we first mapped 450k probes onto binding sites of 58 transcription factors (TFs), as assessed by the ENCODE consortium [23] in the H1 hESC line (Methods). The average fraction of 450k probe CpGs mapping within a binding site of one of the 58 TFs was 3% (∼ 16316 probes), with Pol2 exhibiting the largest overlap (12%) and BCL11A the lowest (< 0.01% i.e. less than 454 probes) (**S5_Fig in** S1 Text). Enrichment analysis was then performed for each one of the 58 ENCODE transcription factors, and separately, on the hypermethylated and hypomethylated DMRs (**Table S2 in** S1 Text). Confirming the results of Ziller et al [19], we observed that DMRs which exhibited lower levels of methylation in hESCs were massively enriched for binding sites of well-known pluripotency factors such as POU5F1 and NANOG (Fisher-test *P* < 10^−10^, Fig. 5C). Out of the 58 TFs considered, besides OCT4 and NANOG, only 7 others (BCL11A, HDAC2, SP1, MAFK, c-JUN, CtBP2 and RXRA) showed specific enrichment (all significant with Fisher-test *P* < 10^−5^ and with Benjamini-Hochberg (BH) adjusted *P* < 0.05) among DMRs hypomethylated in hESCs. A total of 5 TFs (TCF12, p300, TEAD4, ATF2, JUN-D) showed bivalent enrichment in both hypermethylated and hypomethylated DMRs. The rest of TFs showed specific enrichment among the DMRs hypomethylated in differentiated cells (Fig. 5C). Notably, CTCF was the most strongly enriched TF among DMRs losing methylation in differentiated cells. It is also noteworthy that components of the polycomb complex (e.g. SUZ12 or EZH2) were not enriched in either the hypermethylated or hypomethylated DMRs (Fig. 5C), suggesting that polycomb binding does not play a major role in cellular differentiation. Confirming the robustness of the results and reliability of the ENCODE data, we observed that ChIP-Seq binding profiles of the same TF but generated by different laboratories had very similar enrichment values (Fig. 5C).

Among the 60 somatic tissue samples, some tissues were represented in sufficient numbers to also allow for tissue-specific analyses. To see whether differential DNA methylation would allow us to infer lineage-specific transcription factors, we considered the case of liver-tissue [19]. Because ChIP-Seq for a reasonable number of TFs (more than 50) has only been performed for a liver cancer cell line (HepG2), we used the binding site profiles as determined in this cell-line. We identified a total of 1547 DMRs hypomethylated in hESCs compared to normal liver samples (n = 4), compared to as many as 10670 DMRs which were hypomethylated in the normal liver cells. TFBS enrichment analysis revealed massive enrichment of FOXA1, FOXA2, CEBPB, HNF4A and HNF4G binding sites (Fisher test *P* < 10^−10^, BH-adjusted *P* < 0.05) among the liver-specific hypomethylated regions (Fig. 5D & **Table S3 in** S1 Text), once again consistent with previous observations [19] and with the previously documented role of these TFs in liver specification [38, 39].

### Differential DNA methylation patterns identify REST and chromatin organization factors as key targets of disruption during aging

**Fig 6:**
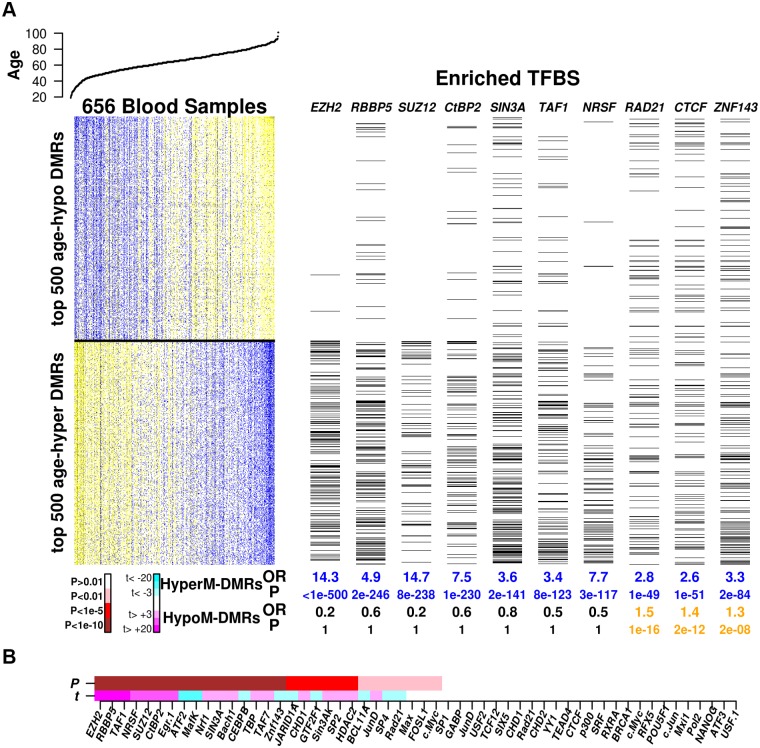
Enrichment of TFBSs among age-DMRs. **A)** Left top panel depicts the age of the individuals from which the blood samples were taken, sorted by increasing age. Heatmap depicts relative DNA methylation levels for the top 500 age-hypomethylated and top 500 age-hypermethylated DMRs (blue = relative high DNA methylation, yellow = relative low DNA methylation), with samples sorted by increasing age. Right panel depicts (with black lines) which DMRs overlap with transcription factor binding sites (TFBS) for a number of TFs which exhibited highly significant enrichment either for age-hypermethylated or age-hypomethylated DMRs (or both). Below the panels we give the odds ratios (OR) and Fisher-test P-values (P) of enrichment of the corresponding TFBSs among age-hypermethylated and age-hypomethylated regions of the top 5% of age-DMRs. **B)** Combinatorial enrichment analysis of TFBSs. TFs have been sorted according to the strength of association (P-value) of their binding profile with the association of a region’s DNA methylation with age, as assessed from a multivariate linear regression model. Color code for P-values: white (*P* > 0.01), pink (*P* < 0.01), red (*P* < 1*e* − 5), brown (*P* < 1*e* − 10). Below P-value bar we show the corresponding estimated t-statistic in the multivariate analysis (magenta = strongly significant (*P* < 0.001) positive values, cyan = strongly significant (*P* < 0.001) negative values).

Having validated our algorithm in the context of cellular development, we next asked if specific TFBSs may be enriched among age-associated DMRs. Using ENCODE TFBSs as assessed in the H1-hESC line, we found that even though only the minority (3036 DMRs, 25%) of age-DMRs were hypermethylated, that these were more strongly enriched (**Table S2 in** S1 Text). The transcription factors most strongly enriched among age-hypermethylated DMRs were two polycomb components (EZH2, SUZ12), as well as RBBP5 and NRSF/REST (Fig. 6A). Far fewer TFs were enriched among age-hypomethylated DMRs, but most of these also showed enrichment of binding sites among age-hypermethylated DMRs (e.g. RAD21, ZNF143, CTCF) (Fig. 6A, **Table S4 in** S1 Text). We note that most of the enrichments were highly significant (Fisher-test *P* < 10^−10^, BH-adjusted *P* < 0.05, Fig. 6A, **Table S4 in** S1 Text).

Because many transcription factors co-bind at specific sites, we next performed a multivariate regression analysis to assess if the association of a given TF binding profile with age-associated DNA methylation is independent of co-binding by other TFs (Methods). We note that no pair of distinct TFs exhibited a Jaccard Coefficient overlap higher than 0.7, thus allowing us to use all distinct TF binding profiles in the multivariate analysis, which largely confirmed the previous enrichment analysis (Fig. 6B). Specifically, the polycomb factors (EZH2, SUZ12) were once again highly enriched among age-hyperM DMRs, as well as *RBBP5, TAF1, NRSF/REST, CTBP2* and *EGR1*. TFs whose binding sites were enriched among age-hypoM DMRs included as before *RAD21* and *ZNF143*, but now also *ATF2, CEBPB* and *JARID1A*. We verified that results were largely unchanged had we used the age-DMRs from the cellular heterogeneity adjusted analysis (**SI, S6_Fig in** S1 Text).

Next, we asked if particular TFs have binding sites enriched within the previously identified age-associated hypomethylated blocks. Not unsurprisingly, this revealed TFs whose binding sites we previously found to be enriched within age-hypoM DMRs (e.g. *RAD21, ZNF143, CTCF*, **S7_Fig in** S1 Text). However, it also identified a number of TFs whose binding sites were enriched only among age-hyperM DMRs, specifically this was the case for *SIN3A, TBP* and *TAF1*. Interestingly, these specific TFs also had substantially more binding sites within open-sea regions, in comparison to say the polycomb factors (*EZH2,SUZ12*) which exhibited the least enrichment of binding sites within blocks (**S7_Fig in** S1 Text).

In summary, our integrative DNA methylation ENCODE analysis not only points towards an age-associated disruption of PRC2 binding, but also of that of important transcription factors like *REST*, which has recently been strongly implicated in Alzheimer’s [40], and *RBBP5*, which interacts with members of the histone methyltransferase MLL complex. In addition, the data points towards a potential binding site redistribution of other transcription factors like *RAD21, ZNF143* and the chromatin organization factor *CTCF*. Of note, pluripotency factors, which were strongly enriched in the previous cellular development/differentiation analysis, were not so in the aging analysis (**Tables S2 & S4 in** S1 Text & Fig. 6).

## Discussion

Given the emerging importance of age-associated epigenetic drift, we decided to conduct an in-depth novel integrative multi-scale analysis of this epigenetic phenomenon in whole blood tissue, the tissue for which the largest data set is available. Our analysis makes a number of important novel observations, whilst also confirming some earlier findings made with the less comprehensive Illumina 27k arrays [41].

First, we have demonstrated that most of the epigenetic drift is not caused by underlying changes in blood cell subtype proportions. Indeed, focusing on the 5% most significant age-associated DNA methylation changes, we observed that over 80% of these changes retained statistical significance in an analysis adjusted for shifts in blood cell subtype proportions. Intriguingly, we observed that age-DMRs were enriched most strongly for CpG island hypermethylation and that this enrichment increased upon adjustment for cellular heterogeneity (Fig. 1). We note that although opensea and shore/shelf probes were generally much more numerous among age-DMRs (Figs. 1B & 1E), that this seems to only reflect the bias of the 450k array towards this class of probes. Normalizing for this bias, as we did here, shows that CGIs have the highest probability of being an age-DMR (Figs. 1A & 1D). The stronger enrichment of CGIs in the adjusted analysis is also consistent with two previous observations: First, that the main age-associated shift in blood cell subtype composition, ie. an increase in the granulocyte to lymphocyte ratio, is accompanied by a global loss of methylation [7], and secondly, that a significant proportion of developmental DMRs are distal to promoter CGIs and TSSs [19]. Thus, if a substantial proportion of the myeloid/lymphoid cell subtype specificity is conveyed by DNA methylation patterns in shore/shelf and open-sea regions, then this would explain why these regions are less enriched after adjustment for the age-associated myeloid/lymphoid skewing. All these are important observations, because it suggests that the mechanisms leading to the age-associated modulation of the DNA methylome are very distinct to those implicated in development and differentiation. This is perhaps not surprising, since otherwise drift, which is already prominent in early life [42, 43], would have a dramatic effect on normal tissue homeostasis well before the normal aging effects become visible [10]. Our integrative analysis with a large blood gene expression data set further supports this view, since we found that drift does not significantly alter *in-cis* gene expression levels, a result which is also consistent with a recent age-related gene expression study conducted in lymphoblastoid cell-lines [44]. In fact, quite remarkably, we observed that drift may act to stabilize pre-existing baseline levels of gene expression. Indeed, we found that age-associated hypermethylation at CGIs preferentially targets genes that are not expressed in blood tissue, whereas age-associated hypomethylation correspondingly targets more highly expressed genes (Figs. 4A & B). This confirms an earlier observation made by Day et al with the older Illumina 27k beadarrays [41], and was further validated in another large gene expression data set (Fig. 4B).

It is important to point out that our observation that a significant proportion of epigenetic drift is not caused by changes in blood-cell type composition does not contradict the observations and recommendations of Jaffe and Irizarry [14]. As shown here, adjustment for blood-cell type composition does remove some of the epigenetic drift attributable to the increase in the granulocyte to lymphocyte ratio (Fig. 1F **& S2_Fig in** S1 Text). Most importantly, however, it should be clear that the effect of cell type compositional changes on inferred DNA methylation patterns will depend on two main factors: the tissue type and the phenotype being considered. Thus, when comparing patterns of DNA methylation in blood tissue between cancer patients and healthy controls, there is a strong need for adjustment because the presence of the tumour induces dramatic changes in blood-cell type composition [12, 14, 15]. Likewise, when comparing DNAm patterns in blood of Rheumatoid Arthritis (RA) patients to that of controls, adjustment is critical as demonstrated by Liu et al [11]. Our data strongly supports the view that age has a much less dramatic effect on changes in blood cell type composition, compared to cancer-presence or RA.

Another important contribution is the demonstration of large megabase-scale blocks of age-associated hypomethylation, covering 14% of the genome, and, importantly, that only a subset of these blocks are enriched for age-associated CGI hypermethylation (Fig. 2, **Table S1 in** S1 Text). Interestingly, we also found that age-associated hypomethylated blocks preferentially undergo hypomethylation in cancer compared to other open sea regions which do not change with age (Fig. 3). However, we also observed some differences in relation to what has been observed in cancer. Notably, in cancer most hypomethylated blocks are enriched for CGI hypermethylation [18]. In contrast, CGI hypermethylation within age-hypomethylated blocks was only seen for a relatively small fraction of blocks. Given that most of the age-associated CGI hypermethylation is independent of blood cell subtype, it is highly plausible that these specific changes are also present in other tissue types. Indeed, that the age-hypermethylated component of drift appears to be specially independent of tissue type was an observation made by us previously with Illumina 27k beadarrays [2] and further confirmed by others [28]. Thus, it is of interest to consider the specific genes targeted for aberrant DNA methylation within these blocks. For instance, we identified a hypomethylated block on chromosome-4, containing age-hypermethylated CGIs targeting the promoter of the *HAND2* gene. This transcription factor has been shown to be causally implicated, through DNAm induced silencing, in the development of endometrial cancer [29]. Specifically *HAND2* mediates the tumour suppressive effects of progesterone [29]. Interestingly, age is also one of the main risk factors for endometrial cancer, hence it is plausible that age-associated *HAND2* promoter methylation, if present in endometrial tissue, could be a contributing factor to endometrial cancer risk. Indeed, there is already prior evidence that *HAND2’s* promoter undergoes age-associated DNA hypermethylation in epithelial tissues [1]. Interestingly, the effect of *HAND2* methylation on endometrial cancer risk is mediated by methylation and silencing of *HAND2* in endometrial stromal *non-immune* cells, increasing paracrine signaling through release of fibroblast growth factors thus sensitizing the endoemtrial epithelial cells to oncogenic estrogen [29]. That the DNA methylation of *HAND2* in cancer tissue is not attributable to immune cell infiltration, is an important observation because it suggests that some of the common hypermethylation seen in aged blood tissue and in cancer tissue may not be due to immune-cell activation and tumour-infiltration of these activated immune cells.

In this work we also performed an integrative analysis of epigenetic drift, as measured with Illumina 450k beadarrays, with ENCODE data, and specifically with ChIP-Seq transcription factor binding site data for a total of 58 TFs as determined in a human embryonic stem cell line. This analysis not only identified specific polycomb factors (SUZ12, EZH2), previously already known to be targeted by age-associated DNA hypermethylation [2, 3], but also several interesting regulatory factors, including *RBBP5, NRSF, SIN3A, TAF1, EGR1* and *CTBP2*, some of which (e.g. *RBBP5*) have not been previously implicated in aging. For instance, *RBBP5* (retinoblastoma binding protein-5), a protein whose role in aging is only implied from homology (JenAge AgeFactDB database) [45], is part of the MLL1/MLL complex, whose role is to methylate/di-methylate lysine-4 of histone H3, which is a tag for epigenetic transcriptional activation. Thus, age-induced impairment of *RBBP5* binding could lead to functional disruption of the MLL complex and hence to loss of H3K4 methylation, a well-known aging effect [46, 47]. Interestingly, *RBBP5* has also been shown to interact with *TAF1, TAF7* and *TBP*, all members of the transcription factor IID (TFIID) multiprotein complex, and all of which had binding sites enriched among age-hyperM DMRs, even in the multivariate analysis (Fig. 6B **& S8_Fig in** S1 Text). Both *TAF1* and *TBP* have entries in the GenAge HAGR database [48], with *TAF1* being essential for cell cycle progression. Another TF with an entry in the GenAge HAGR database is *SIN3A*: interestingly, *SIN3A* has been shown to function in histone deacetylase pathways [49] (**S9_Fig in** S1 Text), as well as in the deacetylation of the *c-MYC* protein, thus contributing to its repression [50]. Of note, *MXI1*, an interacting partner of *SIN3A*, was also enriched in our TFBS analysis (**Table S4 in** S1 Text), although it lost the enrichment in the multivariate analysis (Fig. 6B). Yet another TF with enriched binding sites among age-hyperM DMRs and with an entry in the GenAge database is *EGR1*. This gene is an important regulator of the cell-cycle, with pro-apoptotic functions and acting upstream of *TP53* (**S9_Fig in** S1 Text). Finally, our list also included *NRSF/REST*, which has a role in suppressing genes which promote Alzheimer’s disease [40]. Thus, our data suggests age-associated functional disruption of *REST*, which may allow Alzheimer promoting genes to be expressed. In addition, we observed that *CTCF* was one of a few number of transcription factors whose binding sites were enriched among both age-hypomethylated DMRs, suggesting a global redistribution of chromatin patterns with age. Thus, overall, our integrative analysis points towards an age-associated disruption of DNA binding of transcription factors with important roles in histone deacetylation, histone methylation, chromatin architecture and tumour suppressor pathways. In this regard, it is worth emphasizing some of the key differences with the corresponding integrative ENCODE analysis performed in the context of cellular differentiation. For instance, pluripotency factors such as *NANOG* or *OCT4*. and chromatin factors such as *HDAC2* or *CTCF2*, all of which play key roles in differentiation from hESCs, played a much less significant role in the context of aging, wheras the opposite is true for the polycomb factors *EZH2* and *SUZ12*.

## Conclusions

In summary, this work has shown that age-associated DNA methylation changes seen in blood are largely independent of changes in blood cell type composition, and reflect patterns of change at different genomic length scales which are reminiscent of those seen in cancer. By integrating Illumina 450k with ENCODE data we have furthermore identified a number of candidate key transcription factors whose regulatory potential may be disrupted during aging.

## Methods

### DNA methylation data sets

For the analysis of aging we used one of the largest available data sets, which profiled over 656 whole blood samples using Illumina Infinium 450k beadarrays [5]. Data was downloaded from GEO and subjected to a stringent quality control analysis, including imputation of missing values, type-2 probe bias correction using BMIQ [51], as well as assessment of the sources of inter-sample variation using a Singular Value Decomposition [12]. The top component of variation correlated with Source site of samples (i.e. laboratory sample was processed), plate and ethnicity. Lower ranked components correlated with gender and age. Samples came from 4 different labs, were processed on 9 different plates (indexed here as 1,2,3,5,6,8,9,10,11) and came from 2 different ethnic groups (white caucasian and hispanic) [5].

For the analysis relating to cellular development and differentiation, we downloaded the Illumina 450k data of GSE31848 from the GEO website. This data consisted of a total of 153 samples, encompassing human embryonic stem cell lines, induced pluripotent stem cells, primary cell lines and somatic differentiated samples from a range of different tissue types. Probes with more than 5% missing values across the samples were removed from analysis. Rest of missing data was imputed using the k-nearest neighbour procedure, as implemented in the *impute R*-package [52]. Because of the inherent bias of type-2 probes and our desire to analyse DNA methylation patterns spatially, we also adjusted this data using the BMIQ algorithm [51].

Cross-reactive probes and probes with SNPs in them were kept in the DNA methylation data analysis. We verified that the main results of this work are independent of whether these probes are included or removed.

### ENCODE data

Transcription Factor ChIP-seq Uniform Peaks *.narrowPeak* files were downloaded from the UCSC ENCODE website (http://hgdownload.cse.ucsc.edu/goldenPath/hg19/encodeDCC/wgEncodeAwgTfbsUniform/). This track contains 690 ChIP-seq datasets representing 161 unique regulatory factors (generic and sequence-specific factors). The dataset spans 91 human cell types and various treatment conditions. These datasets were generated by the five ENCODE TFBS ChIP-seq production groups: Broad, Stanford/Yale/UC-Davis/Harvard, HudsonAlpha Institute, University of Texas-Austin and University of Washington, and University of Chicago. Some TFs were generated by more than one group, or selected by different antibodies. In all cases, human Illumina 450K CpGs were mapped to TF binding sites. Only CpGs falling within the ChIP-Seq peak boundaries were considered as defining an overlap. Here we considered mainly two cell-lines: H1-hESC and HepG2. The number of profiled TFs were 58 (H1-hESC) and 75 (HepG2).

### mRNA expression data

To integrate the blood DNA methylation data from Hannum et al, with gene expression, we first used a whole blood data set (233 samples) generated using the Illumina HumanRef-12v3 array [33]. The normalized data was provided at the probe-level. Probes mapping to the same Entrez gene ID were averaged resulting in a 16345×233 gene expression data matrix. This data matrix underwent further quality control using a Singular Value Decomposition (SVD) method [12] to assess the sources of inter-sample variation. We found that the top PC correlated with beadchip and not age. In deriving differentially expressed genes (DEGs) associated with age using a linear regression model, we also observed statistical confounding, as deduced from the shape of the P-value histogram [53]. To address this problem, we identified and ranked age-DEGs using the ISVA-algorithm [53].

As a validation of the gene expression analysis, we also downloaded another relatively large gene expression data of 198 whole samples [36]. This data was generated with Agilent Whole Human Genome Microarrays [36]. The normalized data was provided at the probe-level. Probes mapping to the same Entrez gene ID were averaged resulting in a 19751×198 gene expression data matrix. This data matrix underwent further quality control using a Singular Value Decomposition (SVD) method [12] to assess the sources of inter-sample variation. In deriving differentially expressed genes (DEGs) associated with age using a linear regression model, we adjusted for sex, case/control status and smoking status.

In order to obtain estimates of baseline levels of gene expression in blood we collected an expression data of cord blood and placenta samples [35]. This data was generated with Illumina Human Ref-8 beadarrays [35]. The normalized data was provided at the probe-level. Probes mapping to the same Entrez gene ID were averaged resulting in a 18342×183 gene expression data matrix. This data matrix underwent further quality control using a Singular Value Decomposition (SVD) method [12] to assess the sources of inter-sample variation. In this data set there were 64 cord blood samples, 54 placenta samples and 65 maternal blood samples. We restricted to analysis of cord blood and placenta samples from non-smokers (n = 45 cord blood, n = 37 placenta).

### Age-DMR analysis

To identify age-DMRs in the Hannum et al data [5], we used linear regressions with plate and gender as covariates. Note that we did not include source and ethnicity as covariates because these were fully correlated with plate. Indeed, plates-1, 2, 3, 8, 10 and 11 contained only caucasian samples, specifically their numbers were 90, 84, 69, 81, 17 and 85. The other plates, i.e. plates-5, 6 and 9 only contained hispanics (90, 92 and 48). It follows from this design that adjusting for plate automatically adjusts for ethnicity, but not vice-versa. Similarly, plate and lab were fully correlated, thus adjusting for plate also adjusts for source-site. Moreover, it is well known that adding highly correlated covariates in multivariate regressions may cause singular or near-singular predictor matrices and thus compromise statistical inference. For these reasons, and given that lower ranked SVD components also correlated with gender (gender was equally distributed across plates, with each plate containing males and females), we used as covariates plate and gender.

The linear regressions above were performed at the level of regional probe clusters, following the procedure of Aryee et al [26]. Specifically, 450k probes were first divided into 3 regional classes: open-sea, shore/shelves and CpG Islands (CGIs). We then used the *boundedClusterMaker* function with *maxGap = 500* and *maxClusterWidth = 1500* on probes within each of these 3 classes, separately, resulting in probe clusters within each class. Beta-values for probes within a cluster were averaged, and linear regressions were performed for these averaged values, i.e one linear regression for each probe cluster.Finally, we also remark that we chose a linear model, mainly because previous works have shown how well linear models are able to predict age across a wide range of tissue types [2, 6].

### Correction for cellular heterogeneity

We followed the reference-based procedure of Houseman et al [15], using the R-scripts and reference CpG list provided in that publication. To clarify, we ran the Reference-Based method using reference profiles for CD8+ T-cells, NK-cells, Monocytes, CD4+ T-cells, B-cells and Granulocytes, to infer cell-type proportions in the individual samples. These were subsequently used as covariates in the linear regression analysis.

### Block Age-DMR analysis

In order to see if age-associated DMRs occur on longer length (i.e megabase) scales, we followed the procedure of Aryee et al [26], focusing on the previously constructed regional clusters mapping only to open-sea probes. To define blocks we used the *clusterMaker* function of the *bumphunter* package [26, 27] with *maxGap = 250000* and using the previously constructed regional open sea clusters as input. To find extended DMRs (specifically hypomethylated block regions) we used the *bumphunter* algorithm with *bpSpan = 250000* and 500 bootstrap iterations.

### Association of transcription factor binding sites with age-DMRs

To test enrichment of transcription factor binding sites among age-hypermethylated and age-hypomethylated DMRs we used a one-tailed Fisher’s exact test. However, since two transcription factors can share the same binding sites, we devised a simple mulitvariate framework to assess if the association of a given TF binding site profile with age-DMRs is independent of the other TF binding site profiles. Specifically, denoting by *t*_*r*_ the t-statistic derived from a linear regression of a regional cluster’s (*r*) average DNA methylation level against age, and denoting by *b*_*f*_ the binding site profile of a given transcription factor *f* (so that *b*_*fr*_ = 1 if a binding site of the TF falls within region *r*, *b*_*fr*_ = 0 otherwise), we performed the multivariate regression

$$\begin{array}{c} t=\alpha_{0}+\sum_{f}\;b_{f}\alpha_{f}+\epsilon \end{array}$$ (1)

It follows from standard multivariate regression analysis, that the t-statistics associated with the estimated parameters ${\hat{\alpha }}_{{}_{f}}$ assess the association of any given TF’s binding profile with age-DMRs independently of other TF’s binding profiles.

## Supporting Information

S1 Text The Supplementary Information Text S1 document contains all supplementary figures and tables plus their legends/captions. (PDF)
