# Supplementary material for: An Integrative Multi-scale Analysis of the Dynamic DNA Methylation Landscape in Aging
Source: PLoS Genet. 2015 Feb 18;11(2):e1004996. doi: 10.1371/journal.pgen.1004996 (PMC4334892; doi:10.1371/journal.pgen.1004996)
Supplement: S1 Text — (PDF) [file pgen.1004996.s001.pdf]

Supplementary Information for:

An integrative multi-scale analysis of the dynamic DNA methylation landscape in aging

Yinming Jiao[1,$], Tian Yuan[1,$], Stephan Beck[2], Simone de Jong[3], Roel A. Ophoff [3] and Andrew E. Teschendorff [*,1,4]

[1] CAS-MPG Partner Institute for Computational Biology, Chinese Academy of Sciences, Shanghai Institute for Biological Sciences,320 Yue Yang Road, 200031 Shanghai, China. [2]Medical Genomics Group, Paul O'Gorman Building, UCL Cancer Institute,University College London, 72 Huntley Street, London WC1E 6BT, United Kingdom. [3]Center for Neurobehavioral Genetics, 695Charles E. Young Drive South, 4335B Gonda, Box 951761, Los Angeles, California 90095-1761, USA. [4]Statistical Genomics Group,Paul O'Gorman Building, UCL Cancer Institute, University College London, 72 Huntley Street, London WC1E 6BT, United Kingdom.

[$] Equal Contribution
*Corresponding Author: andrew.teschendorff@ucl.ac.uk

SUPPLEMENTARY FIGURES:

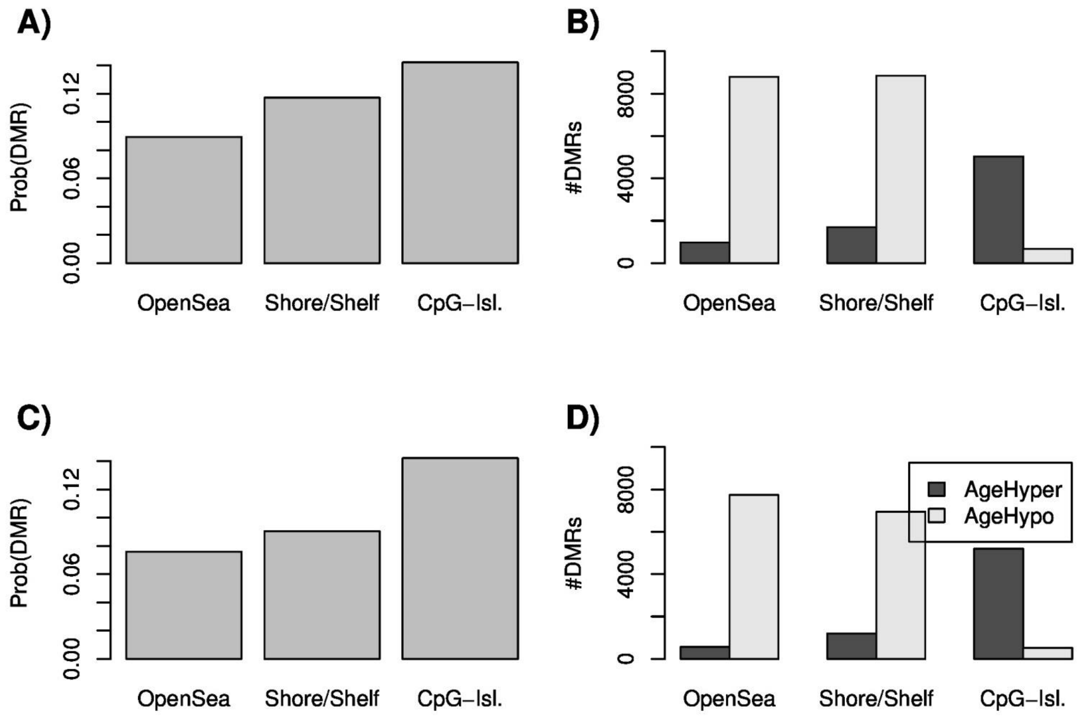

Fig.S1: A) Probabilities that a randomly picked open-sea, shore/shelf and CpG-island region is an age-DMR (defined as those passing the Bonferroni threshold). **B)** Relative numbers of age-hypermethylated and age-hypomethylated DMRs within each regional class. **C)** As A) but now for age-DMRs derived using the reference-based method of Houseman et al, which adjusts for putative changes in blood cell subtype proportions. **D)** As B), but now for the RefBased adjusted analysis.

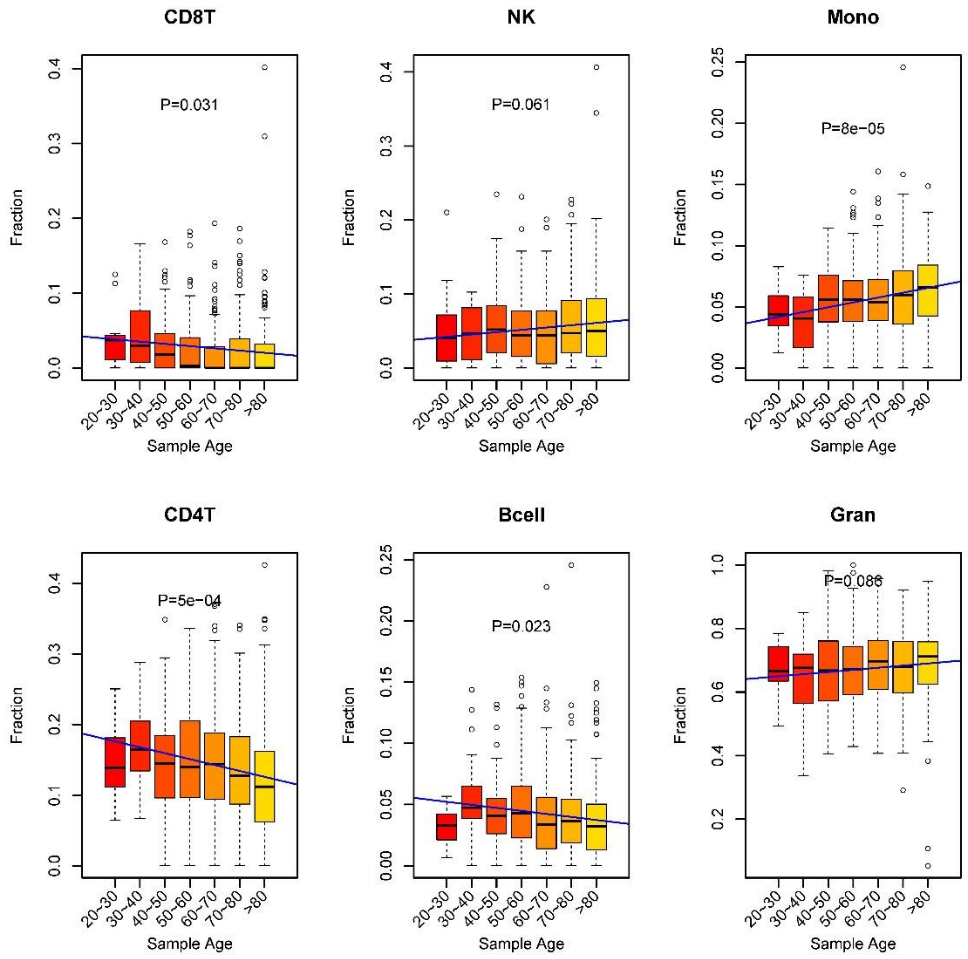

Fig.S2: Boxplots showing the change in cell proportions as a function of age in Hannum et al DNAm data. Each plot represents one blood cell subtype, and samples have been binned into age-groups for ease of visualization. A linear regression line and P-value has been added to each plot.

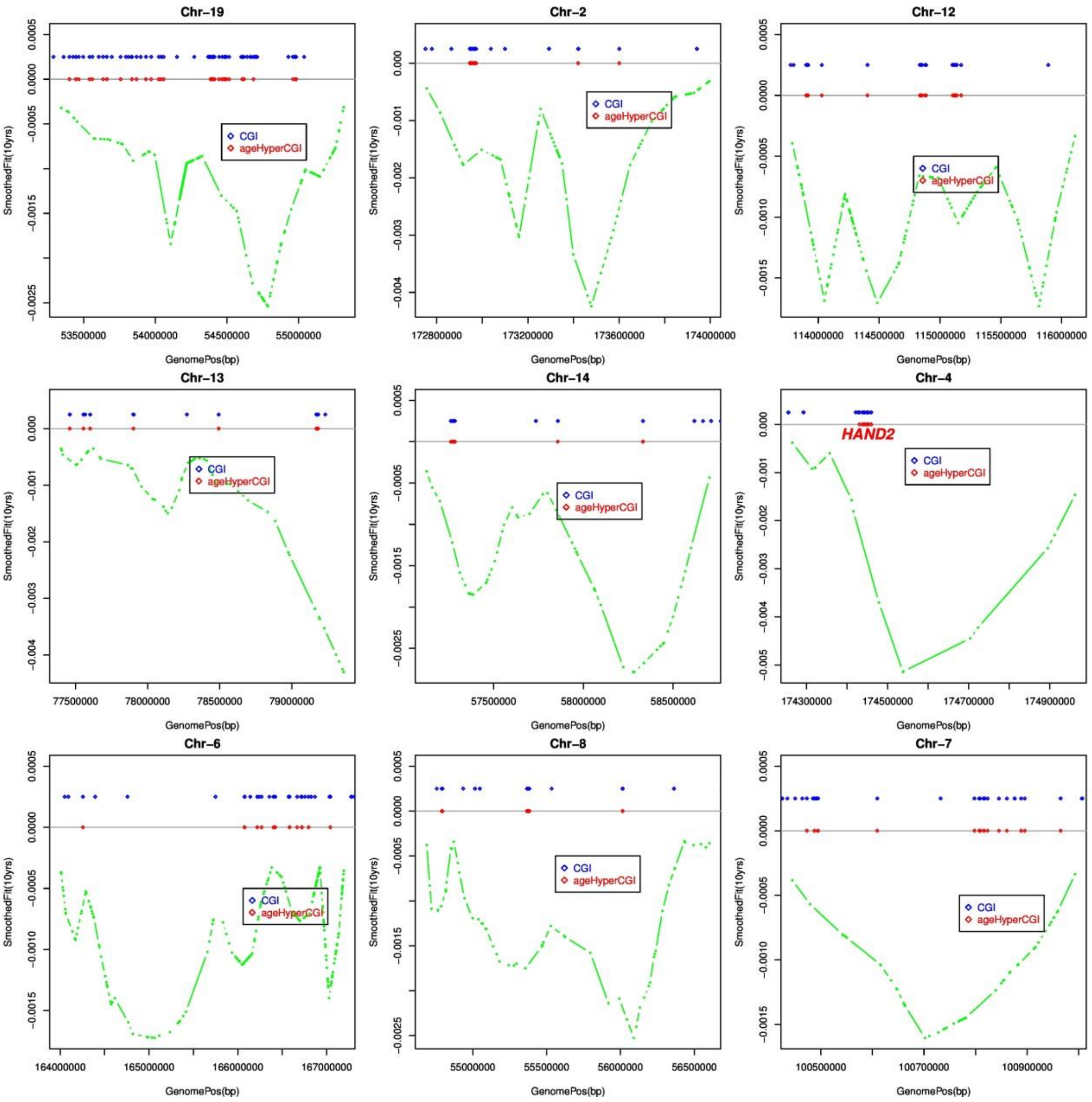

Fig.S3: Nine age-associated hypomethylated blocks, strongly enriched for CGIs undergoing age-associated hypermethylation. Green curve represents the fit of the bumphunter algorithm, indicating the methylation change for an increase of 10 age-years. Blue dots represent CpG islands located within the blocks. Red dots indicate those CpG islands which are significantly hypermethylated. Observe how most blocks contain many well separated age-hypermethylated CGIs, although some blocks (e.g. the one mapping to chr-4 and which contains the *HAND2* gene) are enriched because of many neighboring CGIs becoming hypermethylated.

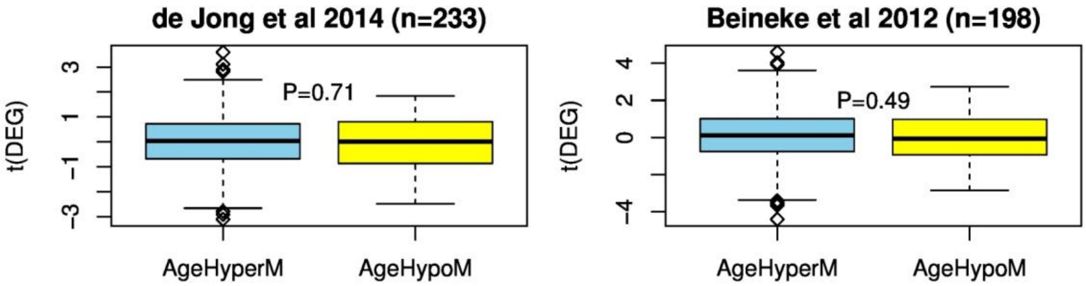

Fig.S4: Statistics of differential gene expression in blood (y-axis) for genes with promoters undergoing significant age-associated hyper or hypomethylation (in blood), respectively. Age-DMRs were restricted to regional probe clusters mapping to CGIs and within 200bp of the TSS or 1st Exon. P-value is from a two-tailed Wilcoxon rank sum test. The blood gene expression data sets are from de Jong et al 2014 and Beineke et al 2012, as indicated , the blood DNA methylation data set is from Hannum et al. (see paper for references).

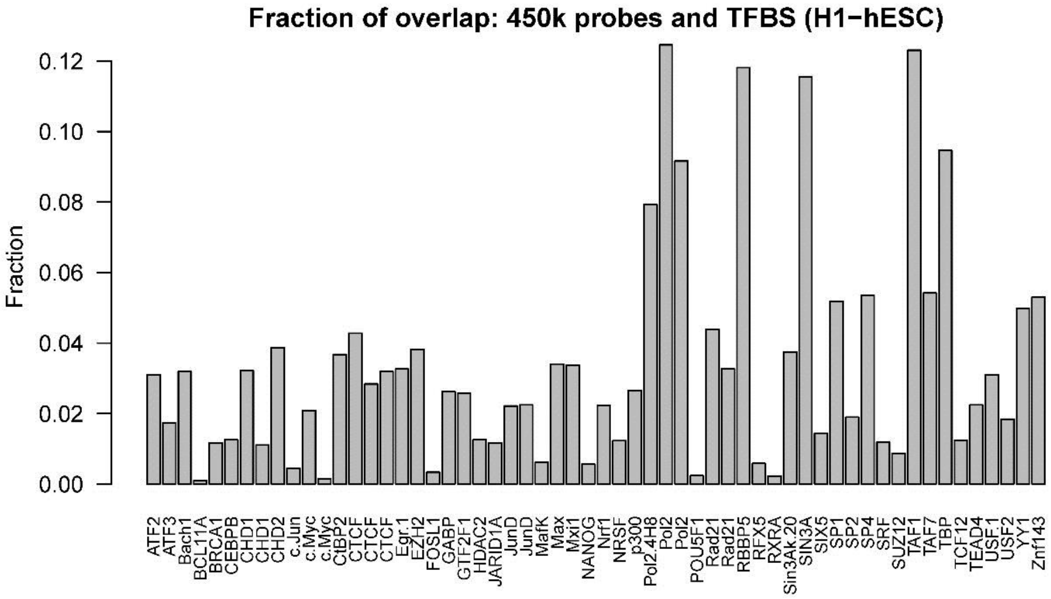

Fig.S5: The average fraction of 450k probe CpGs mapping within a binding site of one of the 58 TFs in H1-HESC.

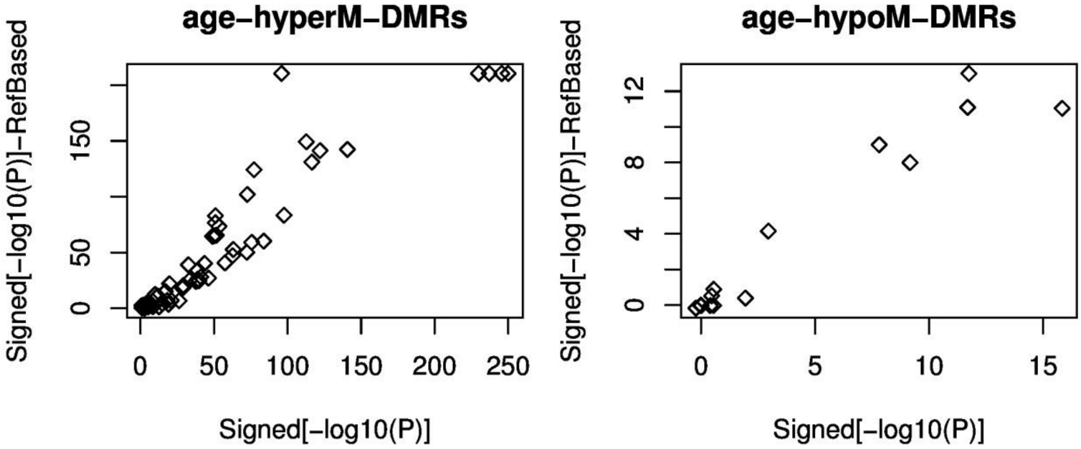

Fig.S6: Scatterplot of the signed –log10 P-values of enrichment between the unadjusted and Reference-based analysis among age-hypermethylated and age-hypomethylated DMRs, as indicated. Observe how transcription factors with enriched binding sites among the age-DMRs in the unadjusted analysis remain generally significantly enriched in the Reference-Based adjusted analysis, suggesting that adjustment for changes in blood cell subtypes does not substantially affect the TFBS enrichment analysis, at least not for the TFs considered here.

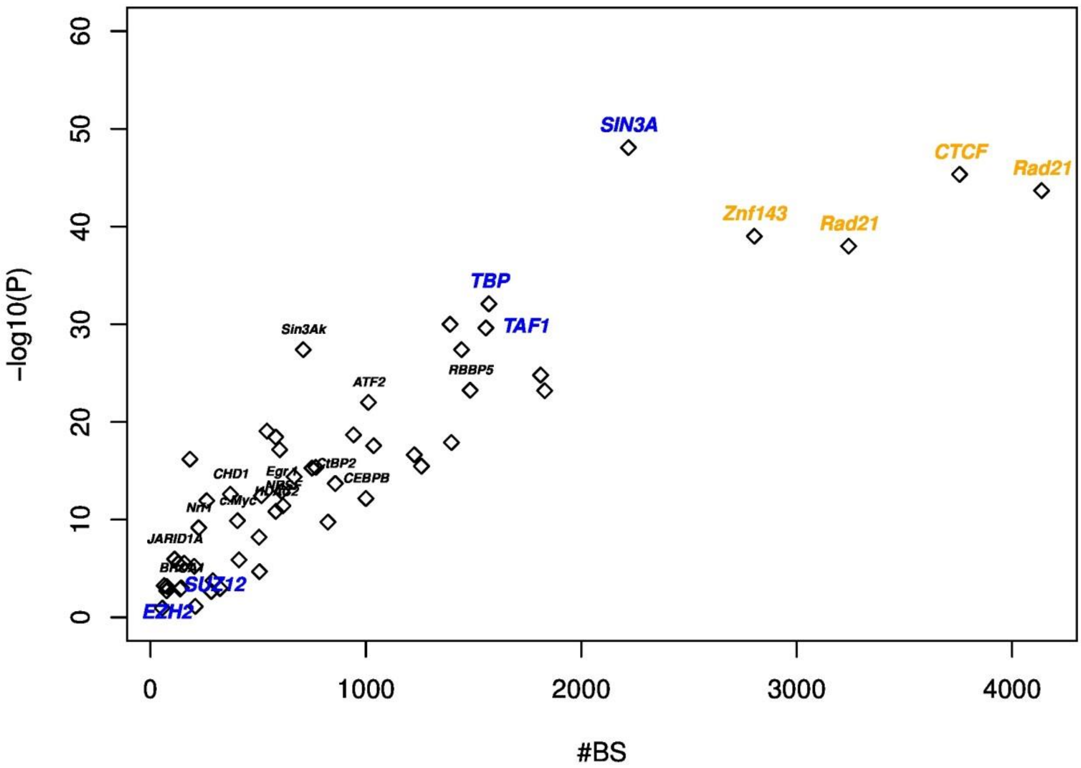

Fig.S7: Scatterplot of –log10 P-values of enrichment of TFBSs within age-associated hypomethylated blocks (y-axis) against the number of binding sites within open sea regions (#BS, x-axis) for TF ChIP-Seq binding profiles as obtained in the H1-hESC line. In blue we indicate some of the TFs with binding sites enriched among the age-hypermethylated DMRs,

whereas in orange we indicate a number of TFs with binding sites enriched among the age-hypomethylated DMRs. Observe how specific TFs like SIN3A or TBP, which have BSs enriched among age-hyperM DMRs, have BSs also enriched within age-associated hypomethylated blocks.

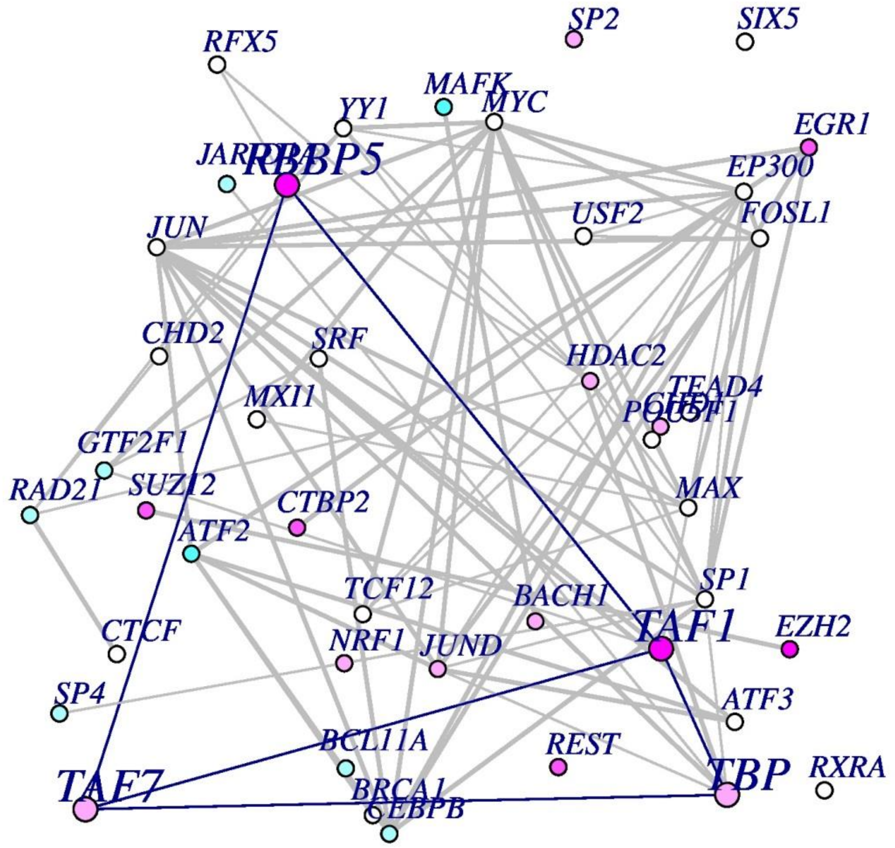

Fig.S8: Mapping of the association of ENCODE transcription factor binding sites (TFBS) with the statistics of age-DMRs, onto a protein interaction subnetwork constructed from the profiled transcription factors. Colors indicate the directionality and strength of the association between a TFBS profile and the corresponding t-statistics of age-associated differential DNA methylation of regions containing this TFBS. t-statistics and P-values were estimated from a multivariate linear regression including all TFs. Color codes: magenta (positive t-statistics and

P<0.001), cyan (negative t-statistics and P<0.001), white (“not significant” i.e P>0.001). Thus, magenta-colored nodes indicate TFs, whose binding sites are more frequent in age-hyperM DMRs, whereas cyan-colored nodes indicate TFs, whose binding sites are more frequent in age-hypoM DMRs, as assessed from a multivariate model, which takes into account co-binding of transcription factors.

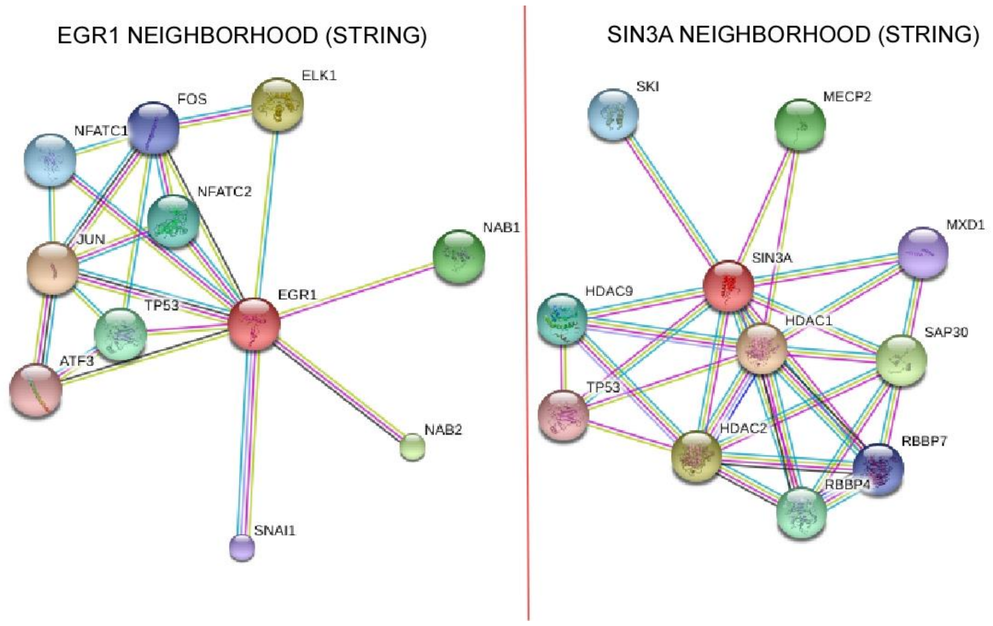

Fig.S9: Protein interaction neighborhoods of EGR1 and SIN3A, two transcription factors with binding sites enriched among age-hyperM DMRs, as derived from the STRING database.

SUPPLEMENTARY TABLES

CHR	Start	End	Dir.	FWER	Obs(AgeCGI)	Exp(AgeCGI)	P
6	28875360	32609783	-1	0	33	31.4	0.324
11	55028287	58612833	-1	0	10	10.76	0.527
11	1542355	2566193	-1	0	16	17.73	0.629
16	62700011	67581437	-1	0	36	28.2	0.034
12	54582926	57499258	-1	0	12	20.06	0.981
19	53342580	55314814	-1	0	38	24.42	6.00E-04
6	32757870	33914814	-1	0	13	13.37	0.475
12	47665230	50720139	-1	0	30	31.11	0.546
3	1.24E+08	1.27E+08	-1	0	16	17.73	0.629
1	1.58E+08	1.61E+08	-1	0	3	6.11	NA
5	1.49E+08	1.51E+08	-1	0	8	9.59	0.654
13	53216641	55818935	-1	0	7	4.36	0.042
17	36553434	38545021	-1	0	34	30.53	0.195

17	31437649	34450758	-1	0	15	13.08	0.211
6	53493997	56792931	-1	0	8	4.65	0.021
11	46998965	48510647	-1	0	7	9.59	0.786
12	1.08E+08	1.1E+08	-1	0	9	8.72	0.368
7	1.42E+08	1.44E+08	-1	0	8	5.23	0.05
10	1.11E+08	1.12E+08	-1	0	2	6.4	NA
17	42741423	44648385	-1	0	20	23.84	0.79
13	1.05E+08	1.09E+08	-1	0	10	4.65	0.001
1	32566833	35206233	-1	0	20	17.73	0.216
15	89987792	92307847	-1	0	15	18.61	0.802
14	69134577	70369903	-1	0	14	10.47	0.073
8	36347870	38068205	-1	0	12	9.01	0.087
5	1.58E+08	1.6E+08	-1	0	13	7.56	0.007
14	1.06E+08	1.07E+08	-1	0	7	16.28	0.997
15	67786689	69318746	-1	0	16	13.37	0.155
2	1.73E+08	1.74E+08	-1	0	16	9.01	0.002
16	57080116	57924256	-1	0	4	5.81	NA
2	2.22E+08	2.24E+08	-1	0	12	6.69	0.005
2	1.37E+08	1.39E+08	-1	0	2	0.58	NA
5	32461615	34053399	-1	0	3	4.36	NA
11	59659903	60710273	-1	0	0	3.49	NA
17	72244448	72957135	-1	0	7	10.47	0.863
2	55237421	56318526	-1	0	2	3.2	NA
8	15215850	16998640	-1	0	4	1.16	NA
6	15256907	15512656	-1	0	0	0.58	NA
4	26024198	26609417	-1	0	3	1.45	NA
6	1.33E+08	1.34E+08	-1	0	3	1.16	NA
3	1.59E+08	1.6E+08	-1	0	5	3.2	0.068
22	23467005	25343683	-1	0	10	13.66	0.846
2	2.08E+08	2.08E+08	-1	0	0	0.87	NA
10	73936754	74804603	-1	0	0	3.2	NA
11	25623877	27303972	-1	0	1	0.29	NA
6	6811759	7641144	-1	0	3	6.11	NA
8	68334606	70372625	-1	0	3	0.87	NA
3	1.16E+08	1.18E+08	-1	0	1	0.29	NA
20	29844546	30804997	-1	0	9	10.18	0.589
12	88178303	89413433	-1	0	3	1.16	NA
10	54203646	55750813	-1	0	0	0.29	NA
2	16790217	16955374	-1	0	0	0	NA
5	90369235	90635839	-1	0	0	0.29	NA
4	1.15E+08	1.16E+08	-1	0	5	1.74	6.00E-04
5	10233474	10663596	-1	0	4	4.07	NA
3	75411042	77214150	-1	0	9	5.81	0.039
9	35510933	36843876	-1	0	4	10.47	NA

1	1.08E+08	1.09E+08	-1	0	2	2.62	NA
4	1.1E+08	1.1E+08	-1	0	2	0.58	NA
14	22951241	23286074	1	0	1	1.16	NA
8	96694077	97549759	-1	0	8	3.78	0.003
11	91450356	92670490	-1	0	5	2.91	0.041
4	62076499	63075650	-1	0	0	0.29	NA
13	80930627	82651186	-1	0	0	0.29	NA
13	37441585	38518217	-1	0	3	1.74	NA
7	93105319	93641451	-1	0	2	0.87	NA
5	75810564	76029522	-1	0	1	0.58	NA
14	99502466	99861110	1	0	5	4.65	0.31
4	69381368	70155747	-1	0	0	0	NA
10	42398529	43216927	-1	0	6	4.94	0.2
14	83560039	83784904	-1	0	0	0	NA
6	98934664	99667847	-1	0	8	4.07	0.007
7	1.24E+08	1.25E+08	-1	0	1	1.45	NA
8	3706763	5028854	-1	0	5	2.33	0.01
20	4680569	5731018	-1	0	4	3.49	NA
4	1.74E+08	1.74E+08	-1	0	0	0	NA
8	73391833	73848687	-1	0	3	0.87	NA
2	91962235	92318652	-1	0	4	2.91	NA
8	33937768	35252359	-1	0	3	0.87	NA
6	1.02E+08	1.04E+08	-1	0	0	0	NA
4	1.75E+08	1.76E+08	-1	0	2	0.58	NA
9	71669714	71999962	-1	0	0	2.33	NA
21	30451483	30713266	-1	0	0	0.29	NA
11	49802549	50371681	-1	0	4	2.62	NA
4	92879355	93646144	-1	0	1	0.58	NA
12	21283725	21670234	-1	0	0	0.58	NA
11	99975877	1E+08	-1	0	0	0	NA
3	63955727	67291183	-1	0.002	8	5.52	0.07
4	1.88E+08	1.91E+08	-1	0.002	12	13.66	0.638
12	1.14E+08	1.16E+08	-1	0.002	38	14.83	3.00E-12
9	1.27E+08	1.29E+08	-1	0.002	17	22.97	0.915
11	1.23E+08	1.23E+08	-1	0.002	6	4.07	0.081
13	77399130	79360870	-1	0.002	11	6.98	0.025
14	57127077	58702245	-1	0.002	12	5.23	2.00E-04
6	71394664	72396934	-1	0.002	7	2.91	0.001
2	50055492	51259931	-1	0.002	2	1.16	NA
13	51326803	51844375	-1	0.002	2	1.45	NA
4	93930622	95380105	-1	0.002	3	1.45	NA
12	93328735	93820263	-1	0.002	0	0.58	NA
4	1.11E+08	1.13E+08	-1	0.002	11	6.4	0.011
8	89218697	89777219	-1	0.002	1	0.29	NA

3	78860887	79416702	-1	0.002	2	0.58	NA
4	1.63E+08	1.64E+08	-1	0.002	4	1.45	NA
11	1.02E+08	1.02E+08	-1	0.002	1	0.87	NA
9	78253033	79306054	-1	0.002	1	2.91	NA
4	1.74E+08	1.75E+08	-1	0.002	10	5.52	0.008
12	97924358	98178057	-1	0.002	0	0	NA
3	81540527	81913557	-1	0.002	0	0.29	NA
22	44963721	45277475	-1	0.002	1	2.62	NA
6	98117205	98565945	-1	0.002	0	0	NA
8	63662874	63921966	-1	0.002	0	0	NA
8	82510379	82784211	-1	0.002	1	1.45	NA
14	47513774	48095881	-1	0.002	0	0	NA
8	66617288	66743912	1	0.002	0	0	NA
20	54942094	55034071	-1	0.002	0	0.58	NA
1	6094104	8492032	-1	0.004	32	29.07	0.223
8	773837	3429430	-1	0.004	9	35.47	1
6	1.64E+08	1.67E+08	-1	0.004	21	12.79	0.003
15	72495878	75047357	-1	0.004	18	17.15	0.344
1	20716191	22463884	-1	0.004	17	15.99	0.321
14	19745841	21624958	-1	0.004	6	9.89	0.903
1	1.52E+08	1.53E+08	-1	0.004	1	0.58	NA
4	9352827	12252318	-1	0.004	10	6.98	0.061
2	1.32E+08	1.34E+08	-1	0.004	20	13.66	0.017
8	1.26E+08	1.28E+08	-1	0.004	0	1.16	NA
11	20390803	25070714	-1	0.004	9	5.52	0.026
20	43343655	45313224	-1	0.004	32	22.97	0.011
11	1.18E+08	1.19E+08	-1	0.004	14	13.96	0.423
21	35468700	36097651	-1	0.004	4	2.62	NA
8	48038289	48437852	-1	0.004	2	1.45	NA
1	60956045	61479999	-1	0.004	0	0	NA
6	96497737	97457431	-1	0.004	1	1.16	NA
6	91600978	92721782	-1	0.004	0	0	NA
2	1.46E+08	1.47E+08	-1	0.004	0	0	NA
7	54142863	54949472	-1	0.004	5	2.33	0.01
5	6828054	7686199	-1	0.004	5	1.45	NA
8	50837079	51576109	-1	0.004	0	0	NA
2	2.12E+08	2.13E+08	-1	0.004	0	0	NA
8	75860623	76227159	-1	0.004	1	0.29	NA
17	10419499	14427896	-1	0.006	13	10.18	0.11
5	16392700	18972260	-1	0.006	10	4.07	2.00E-04
3	54815664	55824354	-1	0.006	9	3.2	3.00E-05
19	41271256	42470790	-1	0.006	9	13.96	0.926
3	1.08E+08	1.1E+08	-1	0.006	0	0.58	NA
4	1.29E+08	1.3E+08	-1	0.006	6	3.2	0.018

3	1.19E+08	1.2E+08	-1	0.006	5	4.07	0.197
8	61119660	62066624	-1	0.006	5	4.36	0.252
8	1.03E+08	1.04E+08	-1	0.006	0	2.62	NA
19	17386421	18415747	-1	0.006	18	33.73	0.999
4	1.6E+08	1.61E+08	-1	0.006	0	0	NA
9	72978571	73103756	-1	0.006	1	1.74	NA
20	47862976	48178928	-1	0.006	1	1.74	NA
9	19424228	19657248	-1	0.006	0	0	NA
6	69717664	69990372	-1	0.006	0	0	NA
18	21451585	21741492	1	0.006	1	0.87	NA
6	36817048	41176577	-1	0.008	14	15.41	0.601
16	78585546	83992394	-1	0.008	10	9.59	0.356
16	84581885	85654314	-1	0.008	8	12.5	0.914
7	1.36E+08	1.38E+08	-1	0.008	6	4.07	0.081
9	1.37E+08	1.38E+08	-1	0.008	2	7.85	NA
11	1.31E+08	1.32E+08	-1	0.008	2	1.45	NA
2	1.22E+08	1.24E+08	-1	0.008	3	3.2	NA
1	1.47E+08	1.49E+08	-1	0.008	6	9.59	0.885
2	1.71E+08	1.72E+08	-1	0.008	10	7.85	0.132
16	47882395	48720568	-1	0.008	0	1.74	NA
10	33562205	34527486	-1	0.008	1	1.45	NA
8	54687817	56602315	-1	0.008	20	7.85	2.00E-07
2	1.64E+08	1.66E+08	-1	0.008	6	2.04	2.00E-04
19	14333799	15271010	-1	0.008	9	9.89	0.547
8	78923501	80789586	-1	0.008	6	3.2	0.018
2	1.7E+08	1.7E+08	-1	0.008	0	1.16	NA
5	97645526	97949352	-1	0.008	0	0	NA
14	84245709	85292067	-1	0.008	0	0	NA
2	76672386	76975220	-1	0.008	0	0	NA
8	64518293	65152945	-1	0.008	0	0	NA
10	79937018	81034374	-1	0.01	4	3.2	NA
17	39977959	41782011	-1	0.01	17	26.17	0.981
5	14244130	16082260	-1	0.01	4	3.49	NA
8	92647788	94937996	-1	0.01	7	3.49	0.008
3	57080664	58836467	-1	0.01	3	8.14	NA
11	43983977	45179364	-1	0.01	8	5.81	0.096
5	1.73E+08	1.74E+08	-1	0.01	3	2.62	NA
17	48382610	50188449	-1	0.01	6	10.76	0.944
16	24931370	27476265	-1	0.01	4	4.65	NA
19	51529858	52328110	-1	0.01	9	9.01	0.413
12	12753718	13748224	-1	0.01	4	6.4	NA
2	42442880	43372366	-1	0.01	5	3.49	0.104
9	1.36E+08	1.36E+08	-1	0.01	7	12.5	0.959
2	3704839	5749552	-1	0.01	2	1.45	NA

5	41968425	44048305	-1	0.01	6	8.72	0.813
11	89535662	90155977	-1	0.01	1	1.16	NA
5	1.19E+08	1.19E+08	-1	0.01	0	0.29	NA
11	6401965	8264034	-1	0.012	13	9.3	0.055
1	1.74E+08	1.77E+08	-1	0.012	7	4.65	0.063
5	1.41E+08	1.42E+08	-1	0.012	8	8.43	0.477
10	75351076	76866367	-1	0.012	5	7.85	0.84
6	1.56E+08	1.57E+08	-1	0.012	2	1.74	NA
1	81510925	82951578	-1	0.012	3	0.87	NA
21	31538828	32255212	-1	0.012	0	0	NA
15	87204431	88843899	-1	0.012	1	0.87	NA
18	43842240	44644942	-1	0.012	5	4.36	0.252
3	5325634	5625835	-1	0.012	0	0	NA
7	37123550	40819169	-1	0.014	9	6.69	0.101
8	11832525	14710963	-1	0.014	8	6.11	0.126
11	15099930	17278779	-1	0.014	7	6.11	0.246
6	26510580	27706264	1	0.014	12	10.18	0.192
8	29019587	30210405	-1	0.014	0	3.49	NA
5	70670374	71687192	-1	0.014	4	2.04	NA
20	30994076	32156436	-1	0.014	3	6.69	NA
7	75513140	76054527	-1	0.014	7	6.69	0.345
12	89586880	90908677	-1	0.014	4	2.91	NA
2	1.45E+08	1.45E+08	-1	0.014	1	1.45	NA
1	13448242	14113201	-1	0.014	4	2.91	NA
4	1.54E+08	1.54E+08	-1	0.014	0	1.45	NA
5	11791728	12883715	-1	0.014	3	1.16	NA
2	1.18E+08	1.19E+08	-1	0.014	1	1.45	NA
13	61090581	61932410	-1	0.014	0	0	NA
22	17955362	18217141	-1	0.014	1	0.87	NA
1	2.08E+08	2.1E+08	-1	0.016	4	4.65	NA
3	1.77E+08	1.79E+08	-1	0.016	0	0.29	NA
1	2.15E+08	2.17E+08	-1	0.016	4	1.16	NA
17	2699556	3343601	-1	0.016	1	1.45	NA
12	96177524	97346526	-1	0.016	3	4.94	NA
16	58121049	59656293	-1	0.016	6	4.65	0.154
12	32287236	33205701	-1	0.016	2	1.74	NA
16	70955223	71446866	-1	0.016	2	0.87	NA
5	91213312	91715141	-1	0.016	0	0	NA
3	71866097	74761390	-1	0.018	7	4.36	0.042
2	2.17E+08	2.19E+08	-1	0.018	1	1.45	NA
3	10137658	11617586	-1	0.018	6	6.11	0.412
1	1.63E+08	1.65E+08	-1	0.018	9	2.91	4.00E-06
5	1.38E+08	1.4E+08	-1	0.018	12	16.86	0.899
4	86594376	87945786	-1	0.018	2	1.74	NA

2	20638399	22114272	-1	0.018	3	2.91	NA
14	24845722	27429693	-1	0.018	3	2.33	NA
2	1.62E+08	1.62E+08	-1	0.018	0	0.29	NA
20	58295012	58983807	-1	0.018	2	2.33	NA
1	2.03E+08	2.03E+08	-1	0.02	6	6.11	0.412
17	6327601	7360681	-1	0.02	15	20.93	0.924
7	1.34E+08	1.35E+08	-1	0.02	0	0.58	NA
7	1.06E+08	1.06E+08	-1	0.02	1	2.04	NA
13	34919279	35735300	-1	0.02	0	0	NA
5	87018149	87880065	-1	0.02	2	0.58	NA
19	22953960	22953960	-1	0.02	0	0	NA
12	1.12E+08	1.13E+08	-1	0.022	3	6.11	NA
2	14163676	16035877	-1	0.022	4	1.74	NA
7	70746877	72040216	-1	0.022	4	1.16	NA
3	12655573	13213415	-1	0.022	1	4.94	NA
19	6730061	7007910	-1	0.022	0	2.62	NA
3	20887296	21447688	-1	0.022	0	0	NA
1	1.5E+08	1.51E+08	-1	0.024	8	14.83	0.979
2	2.19E+08	2.19E+08	-1	0.024	4	4.36	NA
1	1.45E+08	1.46E+08	-1	0.024	6	6.11	0.412
3	45942460	46547809	-1	0.024	0	0.87	NA
8	1.38E+08	1.41E+08	-1	0.024	2	0.87	NA
3	1.85E+08	1.86E+08	-1	0.024	5	3.49	0.104
15	33278159	34531380	-1	0.024	1	2.33	NA
8	98774467	99422528	-1	0.024	5	2.91	0.041
3	1.57E+08	1.57E+08	-1	0.024	2	1.74	NA
2	1.12E+08	1.13E+08	-1	0.024	0	0	NA
1	8908794	9635799	-1	0.026	7	5.52	0.159
14	77079351	78389529	-1	0.026	11	10.76	0.385
8	18411478	19719149	-1	0.026	5	3.2	0.068
15	92849000	93790914	-1	0.026	5	5.52	0.49
8	39695883	40958085	-1	0.026	1	0.29	NA
17	61600386	62401764	-1	0.026	5	6.98	0.74
7	45847100	46754068	-1	0.026	3	0.87	NA
12	1.19E+08	1.2E+08	-1	0.026	6	2.91	0.009
6	23002396	23523214	-1	0.026	0	0	NA
4	1.31E+08	1.31E+08	-1	0.026	0	0	NA
12	61806201	62108676	-1	0.026	0	0	NA
10	38119027	38499434	-1	0.026	0	1.16	NA
9	87023997	87636383	-1	0.026	2	1.45	NA
8	6607096	7754234	-1	0.028	5	3.2	0.068
8	1.34E+08	1.35E+08	-1	0.028	3	2.04	NA
10	86863762	89130195	-1	0.028	7	12.21	0.951
2	11863086	13879237	-1	0.028	3	1.74	NA

7	26341566	27565815	-1	0.028	25	18.32	0.026
2	1.51E+08	1.51E+08	-1	0.028	2	0.87	NA
12	3353356	4598353	-1	0.03	10	7.56	0.104
19	42734510	44218746	-1	0.03	7	14.83	0.991
12	99124116	1.01E+08	-1	0.03	2	2.04	NA
1	1.61E+08	1.61E+08	-1	0.03	3	4.94	NA
1	57514244	58472821	-1	0.03	3	0.87	NA
2	27264194	27505515	-1	0.03	6	6.11	0.412
3	56157811	56896467	-1	0.03	4	2.04	NA
9	98995607	99895750	-1	0.03	4	9.3	NA
9	80073082	80335274	-1	0.03	0	0.58	NA
6	58141967	58263157	1	0.03	1	0.29	NA
12	5127222	6691820	-1	0.032	8	9.01	0.57
2	2.4E+08	2.42E+08	-1	0.032	10	18.61	0.99
7	99440199	1E+08	-1	0.032	4	6.69	NA
11	72581673	73428020	-1	0.032	3	5.23	NA
19	38899811	40276380	-1	0.032	19	25	0.907
10	18133438	19778008	-1	0.032	2	1.45	NA
5	27036352	27120949	-1	0.032	0	0	NA
14	46791389	47311141	-1	0.032	0	0.29	NA
19	22141063	22149119	-1	0.032	0	0	NA
1	2205600	3289902	-1	0.034	14	31.11	1
11	12065669	13205282	-1	0.034	6	3.2	0.018
11	1.12E+08	1.13E+08	-1	0.034	2	5.52	NA
14	21820190	22939357	-1	0.034	1	1.45	NA
7	1E+08	1.01E+08	-1	0.034	17	9.01	7.00E-04
10	1.28E+08	1.28E+08	-1	0.034	2	0.87	NA
12	1.04E+08	1.05E+08	-1	0.036	4	6.4	NA
12	1.17E+08	1.17E+08	-1	0.036	3	3.2	NA
5	39665664	41587899	-1	0.036	2	3.2	NA
4	70860724	71587748	-1	0.036	3	1.16	NA
9	33623433	35042369	-1	0.036	9	14.54	0.946
13	58655819	60055857	-1	0.036	0	0	NA
8	58788163	59366127	-1	0.036	2	1.74	NA
2	2.25E+08	2.25E+08	-1	0.036	0	0	NA
8	65936215	66581126	-1	0.036	0	0.58	NA
1	15598865	16820353	-1	0.038	8	13.66	0.956
11	1.25E+08	1.26E+08	-1	0.038	4	4.07	NA
1	2.37E+08	2.39E+08	-1	0.038	2	1.16	NA
3	1.36E+08	1.37E+08	-1	0.038	2	1.45	NA
11	28487617	29358540	-1	0.038	0	0	NA
4	1.56E+08	1.57E+08	-1	0.038	3	1.45	NA
8	1.19E+08	1.21E+08	-1	0.04	6	2.04	2.00E-04
17	38907446	39306376	-1	0.04	0	0.87	NA

7	77976102	78982418	-1	0.04	0	0	NA
5	79070077	79542470	1	0.04	2	0.87	NA
4	1.6E+08	1.6E+08	-1	0.04	1	1.74	NA
5	1.35E+08	1.38E+08	-1	0.042	10	9.01	0.272
11	1.14E+08	1.15E+08	-1	0.042	2	2.62	NA
3	37332133	38529102	-1	0.042	5	6.69	0.698
16	68691561	69660388	-1	0.042	4	5.81	NA
1	91989267	92907368	-1	0.042	1	2.91	NA
5	91970207	92868380	-1	0.042	0	0	NA
2	1.68E+08	1.69E+08	-1	0.042	3	0.87	NA
8	70421379	70964123	-1	0.042	3	1.45	NA
8	87479250	87721838	-1	0.042	0	0.87	NA
15	62411575	63011250	-1	0.044	3	2.62	NA
3	1.56E+08	1.56E+08	-1	0.044	1	1.74	NA
13	98044144	98893091	-1	0.044	3	3.78	NA
3	1.5E+08	1.51E+08	-1	0.044	4	3.49	NA
10	24909511	25396009	-1	0.044	2	2.04	NA
1	2.12E+08	2.13E+08	-1	0.046	4	6.98	NA
12	1.21E+08	1.22E+08	-1	0.046	3	6.11	NA
10	43656166	44090827	-1	0.046	5	5.23	0.431
6	99857406	99969075	-1	0.046	0	0.58	NA
14	39308020	39519326	-1	0.046	0	0	NA
16	33783802	33956592	1	0.046	4	2.62	NA
1	44047427	46157389	-1	0.048	18	18.32	0.472
10	99928067	1.02E+08	-1	0.048	9	9.01	0.413
11	1.26E+08	1.28E+08	-1	0.048	2	0.58	NA
7	1.46E+08	1.48E+08	-1	0.048	4	1.16	NA
4	67950111	68586568	-1	0.048	1	0.87	NA
5	85577155	86205515	-1	0.048	0	0	NA
5	27387958	27532684	-1	0.048	0	0	NA

Table S1: Table lists the 351 significantly age-associated blocks (FWER<0.05). Columns label the chromosome, approximate start and end positions, (Dir) whether hyper (1) or hypomethylated (1), the Family-Wise Error Rate (FWER), the observed number of age-hypermethylated CGI DMRs contained within the age-associated block, the number expected by the null distribution, and the P-value of enrichment of age-hyperM CGIs within the age-associated block. Some FWERs are indicated as “0” whenever these were <0.001. In the case of the enrichment of age-hypermethylated CGIs, P-values were only estimated for blocks with at least 5 observed age-hypermethylated CGIs, hence the NAs.

TF	OR(hypoM-hESC)	P(hypoM-hESC)	OR(hypoM-DIFF)	P(hypoM-DIFF)
ATF2_SC.81188_None__HudsonAlpha	2.24	4.00E-16	1.69	2.00E-14
ATF3_None__HudsonAlpha	0.38	1	2.04	1.00E-19
Bach1_sc.14700_None__Stanford	0.68	0.999	2	2.00E-35

BCL11A_None__HudsonAlpha	18.01	2.00E-51	0.43	0.989
BRCA1_A300.000A_None__Stanford	0.24	1	1.54	7.00E-05
CEBPB_None__Stanford	1.19	0.132	1.71	6.00E-11
CHD1_A301.218A_None__Broad	0.81	0.975	0.95	0.77
CHD1_A301.218A_None__Stanford	0.78	0.901	0.75	0.988
CHD2_AB68301_None__Stanford	0.57	1	1.31	2.00E-05
c.Jun_None__Stanford	2.85	1.00E-07	1.12	0.27
c.Myc_None__Stanford	0.46	1	1.97	1.00E-20
c.Myc_None__UT.A	0.79	0.731	1.44	0.125
CtBP2_None__USC	1.56	1.00E-06	0.98	0.648
CTCF_None__Broad	1.02	0.402	3.84	<1E-500
CTCF_None__UT.A	0.81	0.983	3.46	3.00E-196
CTCF_SC.5916_None__HudsonAlpha	0.93	0.799	4.06	9.00E-288
Egr.1_None__HudsonAlpha	0.6	1	1.28	7.00E-05
EZH2_39875_None__Broad	0.4	1	0.72	1
FOSL1_SC.183_None__HudsonAlpha	0.42	0.985	1.26	0.124
GABP_None__HudsonAlpha	0.28	1	2.72	5.00E-62
GTF2F1_AB28179_None__Stanford	0.54	1	1.34	2.00E-04
HDAC2_SC.6296_None__HudsonAlpha	3.09	1.00E-23	1.44	3.00E-05
JARID1A_ab26049_None__Broad	0.04	1	1.27	0.028
JunD_None__HudsonAlpha	1.28	0.022	1.23	0.004
JunD_None__Stanford	1.79	6.00E-08	2.06	8.00E-28
MafK_ab50322_None__Stanford	2.43	1.00E-08	1.54	1.00E-04
Max_None__USC	1.07	0.282	2.14	7.00E-45
Mxi1_AF4185_None__Stanford	0.4	1	1.8	2.00E-21
NANOG_SC.33759_None__HudsonAlpha	8.71	1.00E-75	1.11	0.24
Nrf1_None__Stanford	0.04	1	1.2	0.014
NRSF_None__HudsonAlpha	0.44	1	2.05	5.00E-19
p300_None__HudsonAlpha	2.75	4.00E-29	1.64	1.00E-13
Pol2.4H8_None__HudsonAlpha	0.62	1	1.04	0.226
Pol2_None__HudsonAlpha	0.52	1	1.24	6.00E-08
Pol2_None__UT.A	0.45	1	1.19	1.00E-04
POU5F1_SC.9081_None__HudsonAlpha	15.94	2.00E-88	0.5	0.997
Rad21_None__HudsonAlpha	1.18	0.013	4.17	<1E-500
Rad21_None__Stanford	1.04	0.33	4.59	<1E-500
RBBP5_A300.109A_None__Broad	0.6	1	1.23	1.00E-07
RFX5_200.401.194_None__Stanford	0.48	0.985	1.74	2.00E-04
RXRA_None__HudsonAlpha	3.06	9.00E-06	1.69	0.005
Sin3Ak.20_None__HudsonAlpha	0.46	1	1.53	3.00E-13
SIN3A_NB600.1263_None__Stanford	0.52	1	1.92	1.00E-77
SIX5_None__HudsonAlpha	0.16	1	1.39	8.00E-04

SP1_None__HudsonAlpha	1.84	9.00E-15	1.25	3.00E-05
SP2_SC.643_None__HudsonAlpha	0.21	1	1.49	3.00E-05
SP4_V.20_None__HudsonAlpha	0.5	1	1.88	7.00E-30
SRF_None__HudsonAlpha	0.35	1	1.45	3.00E-04
SUZ12_None__USC	0.59	0.994	0.48	1
TAF1_None__HudsonAlpha	0.52	1	1.28	1.00E-10
TAF7_SC.101167_None__HudsonAlpha	0.58	1	1.21	3.00E-04
TBP_None__Stanford	0.62	1	1.3	3.00E-10
TCF12_None__HudsonAlpha	3.51	1.00E-31	1.93	1.00E-15
TEAD4_SC.101184_None__HudsonAlpha	2.21	6.00E-18	1.7	3.00E-17
USF.1_None__HudsonAlpha	0.84	0.947	1.76	8.00E-25
USF2_None__Stanford	0.63	0.998	1.64	2.00E-10
YY1_SC.281_None__HudsonAlpha	1.19	0.025	1.92	3.00E-44
Znf143_16618.1.AP_None__Stanford	0.77	0.998	4.1	<1E-500

Table S2: Table lists the Odds Ratios (OR) and P-values of enrichment (Fisher-test) of transcription factor binding sites, as obtained in the H1-hESC line, for two different classes of DMRs: those hypomethylated in hESCs compared to somatic/differentiated tissue (hypoM-hESC) and those hypomethylated in differentiated tissue compared to hESCs (hypoM-DIFF).

TF	OR(hypoM-hESC)	P(hypoM-hESC)	OR(hypoM-LIV)	P(hypoM-LIV)
ARID3A_NB100.279_None__Stanford	1.6	0.003	1.32	4.00E-05
ATF3_None__HudsonAlpha	0.85	0.745	0.35	1
BHLHE40_NB100.1800_None__Stanford	0.9	0.76	0.69	1
BHLHE40_None__HudsonAlpha	1.24	0.319	0.66	0.992
BRCA1_A300.000A_None__Stanford	0.22	0.999	0.12	1
CEBPB_forskolin__Stanford	1.27	0.11	1.34	3.00E-05
CEBPB_None__Stanford	1.55	0.003	1.6	7.00E-15
CEBPB_SC.150_None__HudsonAlpha	1.57	0.023	1.93	2.00E-16
CEBPD_SC.636_None__HudsonAlpha	0.72	0.967	0.6	1
CHD2_AB68301_None__Stanford	0.51	0.998	0.27	1
c.Jun_None__Stanford	2.32	2.00E-04	0.95	0.683
c.Myc_None__UT.A	0.37	0.999	0.39	1
COREST_sc.30189_None__Stanford	0.81	0.849	0.52	1
CTCF_None__Broad	1.6	1.00E-05	1.03	0.271
CTCF_None__UT.A	1.69	1.00E-04	0.94	0.852
CTCF_None__UW	1.56	6.00E-05	0.93	0.933
CTCF_SC.5916_None__HudsonAlpha	1.59	3.00E-05	0.88	0.992
ELF1_SC.631_None__HudsonAlpha	0.6	1	0.63	1
ERRA_forskolin__Stanford	2.05	0.079	1.03	0.477
EZH2_39875_None__Broad	1.7	0.004	0.41	1
FOSL2_None__HudsonAlpha	1.56	0.001	0.94	0.852

FOXA1_SC.101058_None__HudsonAlpha	1.68	1.00E-04	1.79	4.00E-27
FOXA1_SC.6553_None__HudsonAlpha	1.76	2.00E-05	1.83	2.00E-29
FOXA2_SC.6554_None__HudsonAlpha	1.77	1.00E-04	1.96	5.00E-31
GABP_None__HudsonAlpha	0.36	1	0.33	1
GRp20_forskolin__Stanford	0.24	0.986	0.2	1
HDAC2_SC.6296_None__HudsonAlpha	1.4	0.01	1.17	0.003
HNF4A_forskolin__Stanford	1.4	0.064	1.62	4.00E-10
HNF4A_SC.8987_None__HudsonAlpha	1.8	8.00E-04	2	3.00E-23
HNF4G_SC.6558_None__HudsonAlpha	1.67	0.002	1.65	4.00E-14
HSF1_forskolin__Stanford	0	1	0.39	1
IRF3_None__Stanford	0.26	0.979	0.11	1
JunD_None__HudsonAlpha	1.16	0.163	0.81	0.999
JunD_None__Stanford	1.81	2.00E-04	1.01	0.482
MafF_M8194_None__Stanford	1.21	0.24	1.81	2.00E-12
MafK_ab50322_None__Stanford	1.41	0.076	1.64	4.00E-09
MafK_SC.477_None__Stanford	1.37	0.109	1.58	1.00E-07
Max_None__Stanford	0.72	0.987	0.45	1
MAZ_ab85725_None__Stanford	0.88	0.835	0.4	1
MBD4_SC.271530_None__HudsonAlpha	1.04	0.472	1.13	0.11
Mxi1_AF4185_None__Stanford	0.74	0.995	0.6	1
MYBL2_SC.81192_None__HudsonAlpha	1.01	0.486	0.99	0.56
NFIC_SC.81335_None__HudsonAlpha	1.42	0.01	1.19	0.002
Nrf1_None__Stanford	0	1	0.05	1
NRSF_None__HudsonAlpha	0.85	0.871	0.3	1
p300_None__HudsonAlpha	1.55	0.002	1.59	7.00E-16
p300_SC.584_None__Stanford	1.52	0.029	0.98	0.594
PGC1A_forskolin__Stanford	0.95	0.604	0.93	0.668
Pol2.4H8_None__HudsonAlpha	0.71	0.998	0.44	1
Pol2_forskolin__Stanford	0.82	0.963	0.38	1
Pol2_None__HudsonAlpha	0.76	0.996	0.46	1
Pol2_None__Stanford	0.84	0.937	0.45	1
Pol2_None__UT.A	0.71	0.997	0.38	1
Pol2phosphoS2_None__Stanford	0.72	0.893	0.36	1
Rad21_None__HudsonAlpha	1.71	1.00E-06	1.09	0.042
Rad21_None__Stanford	1.78	2.00E-06	1.16	0.004
RFX5_200.401.194_None__Stanford	0.59	0.989	0.37	1
RXRA_None__HudsonAlpha	1.78	3.00E-04	1.59	8.00E-12
Sin3Ak.20_None__HudsonAlpha	0.7	0.998	0.52	1
SMC3_ab9263_None__Stanford	1.79	2.00E-05	1.55	4.00E-15
SP1_None__HudsonAlpha	1.11	0.237	1.07	0.099
SP2_SC.643_None__HudsonAlpha	0.38	0.986	0.25	1
SRF_None__HudsonAlpha	0.56	0.966	0.58	1
TAF1_None__HudsonAlpha	0.63	1	0.29	1
TBP_None__Stanford	0.7	0.994	0.48	1

TCF12_None__HudsonAlpha	1.5	0.279	2.3	5.00E-06
TCF7L2_None__USC	2.3	0.051	2.47	6.00E-07
TEAD4_SC.101184_None__HudsonAlpha	2.59	1.00E-09	1.39	1.00E-05
TR4_None__USC	0.33	0.999	0.26	1
USF.1_None__HudsonAlpha	1	0.533	0.88	0.97
USF2_None__Stanford	0.91	0.673	0.7	1
YY1_SC.281_None__HudsonAlpha	0.71	0.997	0.38	1
ZBTB33_None__HudsonAlpha	0.56	0.955	0.25	1
ZBTB7A_SC.34508_None__HudsonAlpha	1.38	0.033	0.63	1
ZNF274_None__USC	0	1	0	1

Table S3: Table lists the Odds Ratios (OR) and P-values of enrichment (Fisher-test) of transcription factor binding sites, as obtained in the HepG2 cell-line, for two different classes of DMRs: those hypomethylated in hESCs compared to liver-tissue (hypoM-hESC) and those hypomethylated in liver tissue compared to hESCs (hypoM-LIV).

TF	OR(hyperM)	P(hyperM)	OR(hypoM)	P(hypoM)
ATF2_SC.81188_None__HudsonAlpha	1.9	4.00E-09	1.03	0.343
ATF3_None__HudsonAlpha	2.62	1.00E-15	0.65	1
Bach1_sc.14700_None__Stanford	3.3	4.00E-51	0.78	1
BCL11A_None__HudsonAlpha	1.78	0.107	1.11	0.392
BRCA1_A300.000A_None__Stanford	3	3.00E-13	0.61	1
CEBPB_None__Stanford	2.1	7.00E-09	1.06	0.283
CHD1_A301.218A_None__Broad	4.35	1.00E-96	0.67	1
CHD1_A301.218A_None__Stanford	3.22	1.00E-17	0.5	1
CHD2_AB68301_None__Stanford	2.71	2.00E-29	0.53	1
c.Jun_None__Stanford	2.75	1.00E-06	0.96	0.604
c.Myc_None__Stanford	2.65	8.00E-19	0.78	0.995
c.Myc_None__UT.A	1.75	0.137	0.19	1
CtBP2_None__USC	7.47	1E-230	0.65	1
CTCF_None__Broad	2.62	1.00E-51	1.37	2.00E-12
CTCF_None__UT.A	2.93	1.00E-51	1.18	0.001
CTCF_SC.5916_None__HudsonAlpha	2.91	4.00E-54	1.36	7.00E-10
Egr.1_None__HudsonAlpha	3.99	3.00E-73	0.51	1
EZH2_39875_None__Broad	14.27	<1E-500	0.22	1
FOSL1_SC.183_None__HudsonAlpha	4.01	1.00E-10	0.5	0.998
GABP_None__HudsonAlpha	3.46	1.00E-41	0.7	1
GTF2F1_AB28179_None__Stanford	2.82	1.00E-21	0.47	1
HDAC2_SC.6296_None__HudsonAlpha	4.77	2.00E-52	0.6	1
JARID1A_ab26049_None__Broad	1.75	0.002	0.36	1
JunD_None__HudsonAlpha	3.37	1.00E-39	0.53	1
JunD_None__Stanford	2.9	6.00E-27	0.87	0.956

MafK_ab50322_None__Stanford	1.65	0.005	1.32	0.011
Max_None__USC	4.54	3.00E-98	0.77	1
Mxi1_AF4185_None__Stanford	3.75	3.00E-58	0.64	1
NANOG_SC.33759_None__HudsonAlpha	3.25	2.00E-12	0.75	0.97
Nrf1_None__Stanford	3.19	6.00E-30	0.38	1
NRSF_None__HudsonAlpha	7.68	3.00E-117	0.52	1
p300_None__HudsonAlpha	3.22	2.00E-38	0.73	1
Pol2.4H8_None__HudsonAlpha	2.35	2.00E-40	0.53	1
Pol2_None__HudsonAlpha	2.55	3.00E-63	0.63	1
Pol2_None__UT.A	2.54	5.00E-47	0.52	1
POU5F1_SC.9081_None__HudsonAlpha	2.64	1.00E-04	0.24	1
Rad21_None__HudsonAlpha	3.07	7.00E-78	1.36	2.00E-12
Rad21_None__Stanford	2.81	1.00E-49	1.5	1.00E-16
RBBP5_A300.109A_None__Broad	4.89	2.00E-246	0.61	1
RFX5_200.401.194_None__Stanford	2.95	2.00E-07	0.97	0.59
RXRA_None__HudsonAlpha	2.25	0.004	1.16	0.277
Sin3Ak.20_None__HudsonAlpha	3.89	3.00E-76	0.59	1
SIN3A_NB600.1263_None__Stanford	3.58	2.00E-141	0.78	1
SIX5_None__HudsonAlpha	1.97	6.00E-06	0.38	1
SP1_None__HudsonAlpha	2.51	5.00E-34	0.6	1
SP2_SC.643_None__HudsonAlpha	2.21	4.00E-08	0.42	1
SP4_V.20_None__HudsonAlpha	2.21	1.00E-19	0.79	1
SRF_None__HudsonAlpha	2	9.00E-06	0.44	1
SUZ12_None__USC	14.74	8.00E-238	0.18	1
TAF1_None__HudsonAlpha	3.37	8.00E-123	0.53	1
TAF7_SC.101167_None__HudsonAlpha	3.44	5.00E-73	0.46	1
TBP_None__Stanford	3.47	2.00E-113	0.56	1
TCF12_None__HudsonAlpha	4.09	1.00E-39	0.69	1
TEAD4_SC.101184_None__HudsonAlpha	2.9	3.00E-33	0.79	0.999
USF.1_None__HudsonAlpha	3	3.00E-44	0.83	0.996
USF2_None__Stanford	2.77	2.00E-20	0.61	1
YY1_SC.281_None__HudsonAlpha	3.14	8.00E-64	0.66	1
Znf143_16618.1.AP_None__Stanford	3.33	2.00E-84	1.3	2.00E-08

Table S4: Table lists the Odds Ratios (OR) and P-values of enrichment of transcription factor binding sites, as obtained in the H1-hESC line, among age-hypermethylated and age-hypomethylated DMRs.
